# A novel plant-fungal association reveals fundamental sRNA and gene expression reprogramming at the onset of symbiosis

**DOI:** 10.1186/s12915-021-01104-2

**Published:** 2021-08-24

**Authors:** Ena Šečić, Silvia Zanini, Daniel Wibberg, Lukas Jelonek, Tobias Busche, Jörn Kalinowski, Sabrine Nasfi, Jennifer Thielmann, Jafargholi Imani, Jens Steinbrenner, Karl-Heinz Kogel

**Affiliations:** 1 Institute of Phytopathology, Centre for BioSystems, Land Use and Nutrition, Justus Liebig University, 35392 Giessen, Germany; 2 Center for Biotechnology - CeBiTec, Bielefeld University, 33615 Bielefeld, Germany; 3 Institute of Bioinformatics and Systems Biology, Justus Liebig University, 35392 Giessen, Germany

**Keywords:** *Brachypodium distachyon*, Genome sequencing, Sebacinalean symbiosis, *Serendipita indica*, Small RNAs

## Abstract

**Background:**

Beneficial associations between plants and microbes are widespread in nature and have been studied extensively in the microbial-dominant environment of the rhizosphere. Such associations are highly advantageous for the organisms involved, benefiting soil microbes by providing them access to plant metabolites, while plant growth and development are enhanced through the promotion of nutrient uptake and/or protection against (a)biotic stresses. While the establishment and maintenance of mutualistic associations have been shown to require genetic and epigenetic reprogramming, as well as an exchange of effector molecules between microbes and plants, whether short RNAs are able to effect such changes is currently unknown. Here, we established an interaction between the model grass species *Brachypodium distachyon* (*Bd*, Pooideae) and the beneficial fungal root endophyte *Serendipita indica* (*Si*, syn. *Piriformospora indica*, Sebacinales) to elucidate RNA interference-based regulatory changes in gene expression and small (s)RNA profiles that occurred during establishment of a Sebacinalean symbiosis.

**Results:**

Colonization of *Bd* roots with *Si* resulted in higher grain yield, confirming the mutualistic character of this interaction. Resequencing of the *Si* genome using the Oxford Nanopore technique, followed by de novo assembly yielded in 57 contigs and 9441 predicted genes, including putative members of several families involved in sRNA production. Transcriptome analysis at an early stage of the mutualistic interaction identified 2963 differentially expressed genes (DEG) in *Si* and 317 in *Bd* line 21-3. The fungal DEGs were largely associated with carbohydrate metabolism, cell wall degradation, and nutrient uptake, while plant DEGs indicated modulation of (a)biotic stress responses and defense pathways. Additionally, 10% of the upregulated fungal DEGs encode candidate protein effectors, including six DELD proteins typical for Sebacinales. Analysis of the global changes in the sRNA profiles of both associated organisms revealed several putative endogenous plant sRNAs expressed during colonization belonging to known micro (mi)RNA families involved in growth and developmental regulation. Among *Bd*- and *Si*-generated sRNAs with putative functions in the interacting organism, we identified transcripts for proteins involved in circadian clock and flowering regulation as well as immunity as potential targets of fungal sRNAs, reflecting the beneficial activity of *Si*.

**Conclusions:**

We detected beneficial effects of *Si* colonization on *Bd* growth and development, and established a novel plant-mutualist interaction model between these organisms. Together, the changes in gene expression and identification of interaction-induced sRNAs in both organisms support sRNA-based regulation of defense responses and plant development in *Bd*, as well as nutrient acquisition and cell growth in *Si*. Our data suggests that a Sebacinalean symbiosis involves reciprocal sRNA targeting of genes during the interaction.

## Background

Mutualistic associations between beneficial microbes and plants are widespread and highly advantageous, especially in the microbial-dominant environment of the rhizosphere. This relationship benefits soil microbes by providing them access to plant metabolites; in return, they enhance plant growth and development by promoting nutrient uptake and/or protection against (a)biotic stresses [1, 2]. The beneficial or parasitic outcome of a plant-microbe interaction is governed by the genotype and physiological status of the host, identity of the microbe, and environmental factors such as soil type and nutrient availability [3, 4]. The establishment and maintenance of mutualistic associations (here called symbiosis) require genetic and epigenetic reprogramming and metabolome modulation by the exchange of effector molecules between the beneficial microbe and the plant [5, 6]. Beneficial microbes have a significant impact on crop production, due to their effects on plant health and yield. However, considerable gaps in knowledge prior to their establishment in agricultural practice remain, including systemic identification of microbial abundance and diversity in various ecosystems, understanding the influence of climate, soil conditions, management practices, and, lastly, elucidating the intricacies of molecular mechanisms governing establishment of colonization and nutrient acquisition [7].

Crucial for regulation of gene expression, RNA interference (RNAi) is a well-known eukaryotic gene silencing mechanism [8], mediated by small RNAs (sRNAs) of 20–24 nucleotides (nt) in size and RNAi-associated proteins, primarily from the Argonaute (AGO), Dicer-like (DCL) and RNA-dependent RNA polymerase (RdRP) families [9]. DCLs generate sRNAs from longer RNA molecules, whereas AGOs bind sRNAs within an RNA-induced silencing complex (RISC). In the context of plant-microbe interactions, microbial protein effectors are known to promote pathogen colonization by suppressing host immune responses [10] and have been described in mutualistic associations as well [5], including the Sebacinalean symbiosis [11]. Recent findings suggest that sRNAs, through RNAi-based regulatory mechanisms, also can serve as effectors of pathogenic microbes [12], whereby the sRNA is secreted to suppress translation of a host mRNA via RNAi. Conversely, plants can secrete sRNAs that target virulence-associated mRNAs in the microbe [13]. This transfer of sRNAs and subsequent gene silencing in the target organism is called cross-kingdom RNAi [12].

We studied the association of the beneficial fungus *Serendipita indica* (*Si*) with the model grass *Brachypodium distachyon* (*Bd*, purple false brome, Pooideae [14];). *Si* is an endophytic fungus belonging to the order Sebacinales that colonizes the rhizodermis and cortex of a large spectrum of plants [15]. *Si* serves as an excellent model for beneficial microbes as it (i) primes plants for disease resistance against biotrophic [16] and necrotrophic [17] fungi, oomycetes [18], and viruses [19]; (ii) enhances the tolerance of plants against abiotic stress [20]; (iii) promotes growth and yield [21]; (iv) has a sequenced 25 Mb genome [22]; and (v) is genetically transformable and culturable in axenic conditions [23]. *Si* initially undergoes a biotrophic growth phase during Arabidopsis (*Arabidopsis thaliana*) and barley (*Hordeum vulgare*) colonization, with suppression of innate immune responses [24, 25] and activation of induced systemic resistance [16]. Subsequently, *Si* colonization of barley enters a cell-death associated phase and switches to a saprophytic lifestyle [26, 27].

*Bd* is a temperate grass species belonging to the Pooideae subfamily and a model for genetic studies of stress resistance and yield parameters of cereals [28]. *Bd* is self-pollinating, genetically transformable, easy to cultivate, and has a sequenced genome of 272 Mb [29–31]. It shares evolutionary proximity and broad synteny with complex crop genomes, such as wheat and rice [14], and is a host for major cereal pathogens [32]. Additionally, RNAi is operational in *Bd*, with proven alteration of micro RNA (miRNA) expression patterns in response to abiotic stresses [33, 34]. In silico analyses revealed that the *Bd* genome, similar to other cereals, contains an expansion of DCL and AGO families [35].

Currently, the significance of cross-kingdom RNAi in mutualistic interactions is largely unknown. A recent in silico study predicted that the arbuscular mycorrhizal fungus *Rhizophagus irregularis* generates sRNAs, which have predicted targets in the host plant *Medicago truncatula* [36]. Moreover, tRNA-derived sRNA fragments from rhizobial bacteria were shown to regulate host nodulation-associated genes by utilizing the host's RNAi machinery [37]. To investigate the role of sRNAs in another agronomically relevant mutualistic interaction, we established a protocol for *Si* colonization of the model Pooideae *Bd*. Additionally, integrative high-throughput sequencing and transcriptome analysis assessed symbiosis-associated changes in the mRNA and sRNA expression patterns of both organisms. We discuss here possible sRNA-based regulation that might be critical for the establishment of the Sebacinalean symbiosis.

## Results

### *Brachypodium distachyon* Bd21-3 forms a mutualistic interaction with *Serendipita indica*

**Fig. 1:**
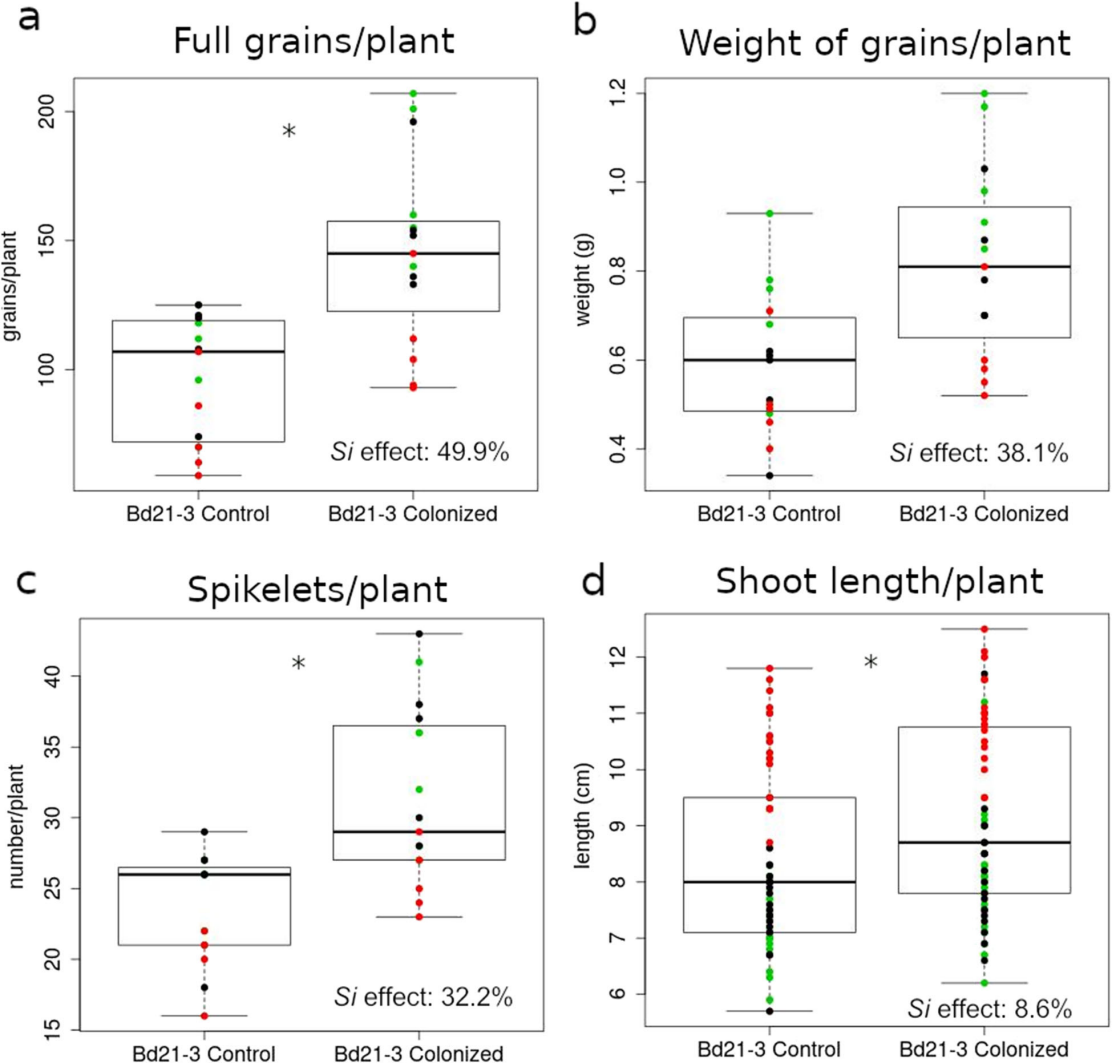
Root colonization by *Serendipita indica* (*Si*) increases growth and yield of *Brachypodium distachyon* (*Bd*) Bd21-3. **a** Number of full grains produced by non-colonized (control) vs. *Si*-colonized plants. Sample size *n* = 5. **b** Total grain weight of control vs. colonized plants. Sample size *n* = 5. **c** Number of spikelets of control vs. colonized plants. Sample size *n* = 5. **d** Shoot length of control vs. colonized plants. Sample size *n* = 20. The results are from three independent biological replicates that are represented by green, black, and red dots on the scatter graph, individual data values for 1a–c in Additional File 1. For **a** and **b**, 1-week-old seedlings were inoculated with 5 × 10^5^ chlamydospores per ml and grown for approximately 3 months in F-E type LD 80 soil; for **c**, spikelets were counted on 2-month-old plants; for **d**, seedlings were grown for 3 weeks on a vermiculite:oil dri mixture (semi-sterile conditions). The significance threshold for *p* values, corrected for multiple testing (Benjamini-Hochberg) was set at 0.05 (*≤ 0.05) and the *Si* effect was calculated as ((Mean_Si-Mean_Control)/Mean_Control) × 10^2^)

To investigate whether *Bd* can develop a beneficial interaction with *Si*, we established an inoculation protocol using one-week-old seedlings of *Bd* line Bd21-3, with dip-inoculation in 5 × 10^5^ chlamydospores ml^−1^ for 3 h. Comparison of grain production in fully mature, colonized vs. non-colonized plants grown in soil showed that *Si* increased the number of filled grains/plant by 49.9% (Fig. 1a, Additional file 1), and total grain weight/plant increased by 38.1% (Fig. 1b, Additional file 1). Consistent with the observation that *Si*-colonized Bd21-3 plants flower several days earlier than control plants, they exhibited a 32.2% increase in the number of spikelets at 2 months after inoculation (Fig. 1c, Additional file 1). Concordantly, growth and biomass analyses of Bd21-3 seedlings revealed a significant 8.6% increase in shoot length (Fig. 1d) upon *Si* colonization.

**Fig. 2:**
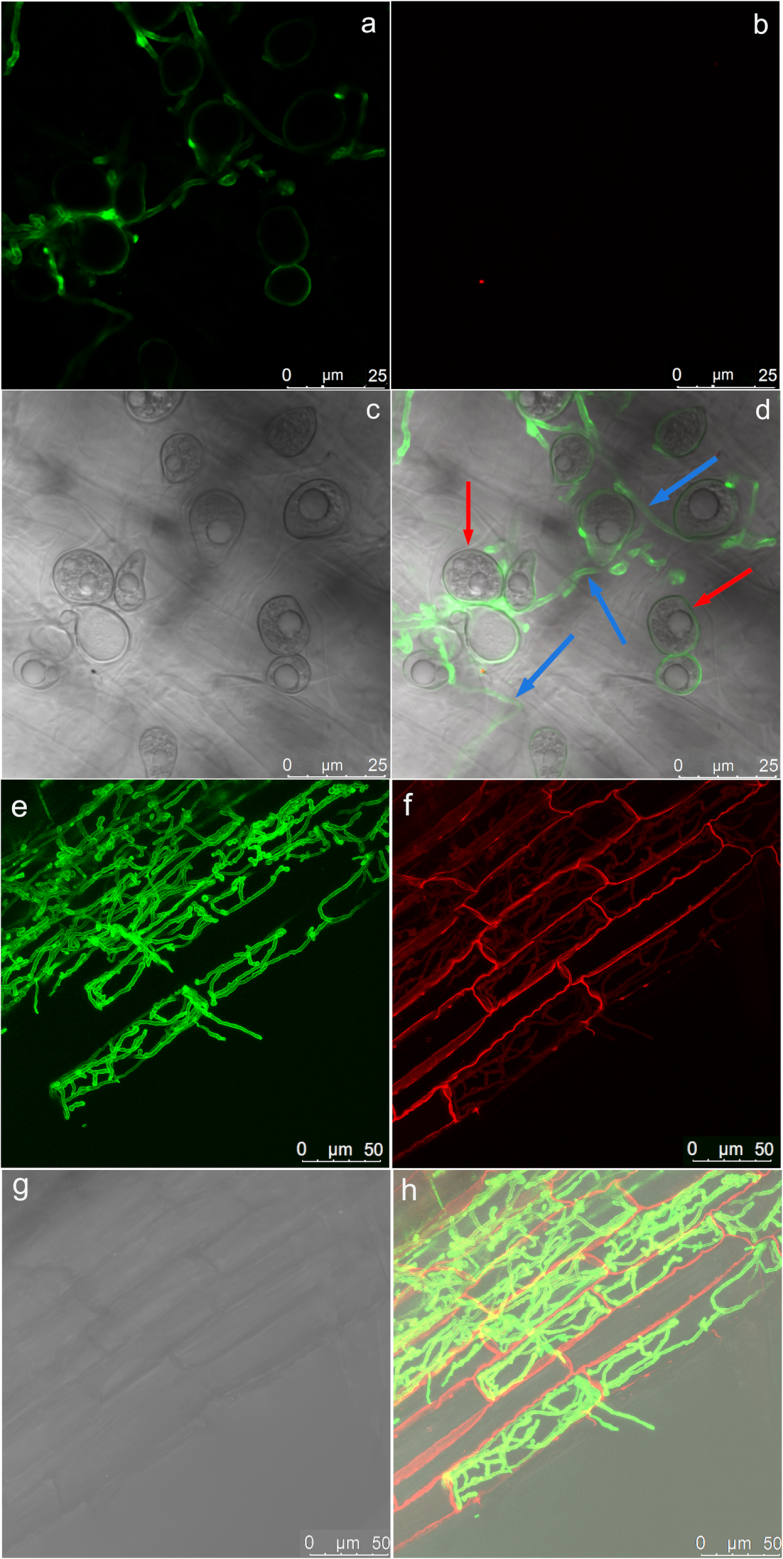
Colonization pattern of *Serendipita indica* (*Si*) on *Brachypodium distachyon* Bd21-3 roots. **a**–**d** Colonization at 4 DPI. **a** Fluorescence microscopy showing WGA-AF488 staining of *Si* cell walls (λexc494 nm, λem515). **b** Fluorescence control (λexc631 nm, λem642). **c** Bright-field microscopy to visualize *Si* chlamydospores. **d** Overlay showing *Si* chlamydospores (red arrows), which have germinated and formed a hyphal network on the root surface (blue arrows). **e**–**h** Rhizodermal root colonization by *Si* at 4 DPI. **e** Fluorescence microscopy showing WGA-AF488 staining of *Si* cell walls (λexc494 nm, λem515). **f** Fluorescence microscopy to visualize propidium iodide staining of root cell walls (λexc535 nm, λem617). **g** Bright-field microscopy to visualize root cells. **h** Overlay showing extensive inter- and intracellular fungal growth on Bd21-3 roots. Imaging was done with a LEICA S8 confocal microscope (e-h: maximum projection; *z*-stack). For **a**–**d**, 1-week-old seedlings were inoculated with 5 × 10^5^ chlamydospores per ml and subsequently grown on a plastic mesh over 0.5X MS; for **e**–**h**, *Si*-inoculated seedlings were grown on vermiculite:oil dri mix before harvesting at 4 DPI

Further analysis of Bd21-3 seedlings indicated that *Si* colonization increased lateral root growth, as early as 4 days post inoculation (4 DPI, Additional file 2: Figure S1a). By 25 DPI, roots showed a more extensively branched structure (Additional file 2: Figure S1b). Microscopy of *Si*-inoculated Bd21-3 roots confirmed root surface colonization and proliferation of fungal spores after staining with chitin-specific WGA-AF 488 at 4 DPI (Fig. 2a–d) and further on at 7 DPI and 14 DPI (Additional file 2: Figure S2). Inter- and intracellular colonization of Bd21-3 cells in the root differentiation zone also was visible after WGA-AF 488 and propidium iodide staining (Fig. 2e–h). These results suggest that establishment of a mutualistic symbiosis correlates with observable phenotypic changes by 4 DPI; thus, this time point was used to further investigate the Bd21-3-*Si* system.

### Resequencing of the *Si* genome

To improve *Si* assembly, the genome was resequenced using MinION (25,167 reads, 324 Mb) and MiSeq (18,225,814 reads, 5.46 Gb); together they yielded approx. 6.0 Gb of sequence information. *De novo* assembly of the Nanopore sequence reads generated 57 contigs, accounting for a total length of 24.7 Mb and a N50 of 1.3 Mb. The draft genome sequence features GC content of 50.8%, similar to the first genome version with 2,359 contigs and GC content of 50.7% [22]. Analyses using the eukaryotic gene prediction tool Genemark-ES 4.33 [38] revealed 9441 predicted genes (75% of the genome; 59,045 exons), 9498 intergenic regions (25% of the genome), and a gene density of 380.68 genes/Mbp (Additional file 2: Table S1). Annotation of the *Si* genes using a GenDB version designed to process eukaryotic genomes possessing multi-exon genes [39, 40] revealed that 4756 have a predicted function. Comparison of predicted genes from the resequenced *Si* genome (Si-2020) vs. the 2011 assembly [22] indicated that the vast majority are shared (90.3%), with 915 genes unique to the Si-2020 genome (Additional file 2: Figure S3). There is a reduction in gene model numbers relative to the 2011 assembly, which can be attributed to improved gene prediction tools for eukaryotic organisms and a considerably reduced number of contigs. Additionally, *Si* shares 2585 genes with another member of the Sebacinales, *Serendipita vermifera*, while 156 genes are shared only with the ectomycorrhizal fungus *Laccaria bicolor* and 2729 genes are common in all three species (Additional file 2: Figure S4).

### Establishment of the *Si*-*Bd* symbiosis is associated with extensive transcriptional reprogramming

**Fig. 3:**
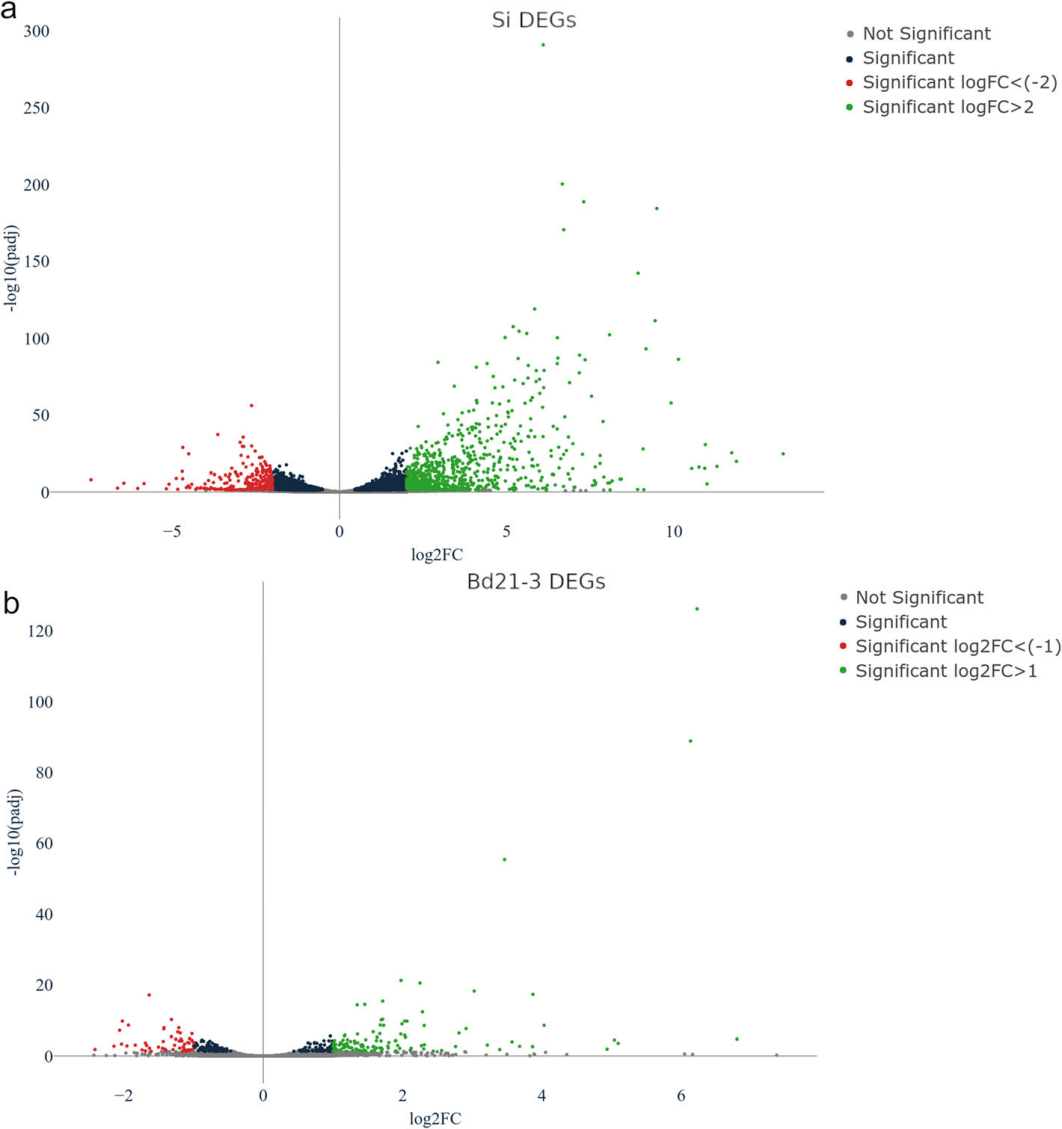
Volcano plots of colonization-associated, differentially expressed genes (DEGs). **a** *Serendipita indica* (*Si*) DEGs identified by comparing reads from colonized roots (Bd-Si) vs. axenic mycelium (Si-ax). **b** *Brachypodium distachyon* Bd21-3 DEGs identified by comparing colonized (Bd-Si) vs. mock-treated (Bd-C) roots. *X*-axis displays the log_2_ FoldChange and *Y*-axis displays the negative log_10_ of adjusted *p* values from DE analysis. The magnitude of up- or downregulation for the DEGs (represented by individual dots) is indicated by different colors, as designated in the legend for each plot

To assess how the symbiotic interaction affects gene expression in both organisms, mRNA was sequenced and analyzed (Additional file 2: Table S2) from the roots of *Si*-colonized Bd21-3 seedlings (sample Bd-Si) and mock-treated plants (Bd-C) at 4 DPI, and from 4-week-old axenic *Si* cultures (Si-ax). Comparison of reads between Bd-Si and Si-ax identified 2963 differentially expressed fungal genes (DEGs, DESeq2: Wald test, Benjamini-Hochberg (BH) adjustment, padj < 0.05), which accounts for 31.4% of the 9441 predicted *Si* genes. Comparison of reads from Bd-Si and Bd-C revealed 317 plant DEGs (0.66% out of approximately 47,917 protein-coding transcripts disclosed in the JGI v1.1 annotation, padj < 0.05). The interaction-responsive DEGs in *Si* and Bd21-3, split into up- and downregulated groups are shown in Fig. 3.

All significant DEGs were submitted to gene ontology term analysis against the reference background for *Bd* and a customized *Si*-specific background. The resulting enriched terms relate to metabolic, mainly redox processes and catalytic activity functions (Additional file 2: Table S3). Of the 25 highly downregulated *Si* DEGs, many encode proteins associated with metabolic reprogramming networks involved in nutrient exchange and adaptation to nutrient availability (Table 1). By contrast, highly upregulated *Si* DEGs encode for proteins involved in fungal catalytic and hydrolytic processes. This suggests that by 4 DPI, *Si* has entered a saprophytic-like growth phase similar to that detected in barley roots [22]. To investigate whether any *Si* DEG encodes effector proteins, a computational pipeline [41] was used to mine 982 genes identified in the Si-2020 genome that encode signal peptide-containing proteins, resulting in 480 putative protein effector genes. In total, 174 (36%) of these were significantly upregulated during colonization of Bd21-3 (Additional file 2: Table S4), including six DELD family proteins [22]. In Bd21-3, many of the highly downregulated interaction-responsive DEGs encode transcription factors or proteins associated with stress responses or circadian clock regulation. Those showing high levels of upregulation include genes linked to immune responses and hormone signaling networks (Table 2 and Additional file 2: Table S3). In order to further validate our sequencing data, we confirmed the expression of five *Si* and five Bd21-3 DEGs from Tables 1 and 2 by RT-qPCR. Generally, the qPCR results show a similar fold change for the selected genes between the colonized root and the respective controls, compared to the mRNA-seq results (Additional file 2: Figure S5). Together, these results show that both organisms utilize a complex enzymatic arsenal to establish and control the symbiosis.

**Table 1:** Top 25 *Serendipita indica* (*Si*) differentially expressed genes (DEGs) during colonization (4 DPI)

Gene	Description	log2FC
1937_g (PIIN_04746)	Related to mismatch base pair and cruciform DNA recognition protein HMP1	− 5.84
465_g (PIIN_02587)	Related to phenylalanine ammonia-lyase	− 5.1
281_g (PIIN_04449)	Probable succinate-fumarate transporter	− 4.7
1121_g (PIIN_02682)	Related to ADY2-protein essential for the acetate permease activity	− 4.68
7809_g (PIIN_07312)	Related to RTM1 protein	− 4.52
2544_g (PIIN_02778)	Probable ADH1-alcohol dehydrogenase I	− 4.42
4482_g (PIIN_08427)	Related to mixed-linked glucanase precursor MLG1	− 4.23
4969_g (PIIN_02119)	Related to meiotic nuclear division protein 1 homolog	− 3.54
1859_g (PIIN_00204)	Probable thioredoxin	− 3.45
5786_g (PIIN_00305)	Probable DHA14-like major facilitator; ABC transporter	− 3.41
4465_g (PIIN_06089)	Putative mitochondrial carnitine O-acetyltransferase	− 3.36
1392_g (PIIN_11719)	Putative alkaline ceramidase 3	− 3.22
8569_g (PIIN_01532)	Related to Ca^2+^-transport (H^+^/Ca^2+^ exchange) protein	− 3.21
2933_g (PIIN_07440)	Related to monocarboxylate transporter 2	− 3.21
758_g (PIIN_02772)	Probable TOM40-mitochondrial import receptor MOM38	− 3.19
2855_g (PIIN_07067)	Related to l-asparaginase	− 3.18
5713_g (PIIN_07616)	Related to MFS transporter	− 3.17
3225_g (PIIN_08230)	Related to RSB1-integral membrane transporter	− 3.11
8602_g (PIIN_08742)	Putative maintenance of mitochondrial morphology protein 1	− 3.09
917_g (PIIN_03155)	Related to YTP1	− 3.05
6928_g (PIIN_00312)	Related to nitrogen metabolic regulation protein	− 2.96
6930_g (PIIN_00314)	Probable malate synthase	− 2.94
4348_g (PIIN_03103)	Putative ubiquitin-conjugating enzyme D4	− 2.9
6400_g (PIIN_07801)	Probable acyl-CoA dehydrogenase short-branched chain precursor	− 2.9
5097_g (PIIN_04235)	Related to acyl-CoA dehydrogenase	− 2.88
5186_g (PIIN_09750)	Probable pectate lyase	11.93
8239_g (PIIN_02110)	Related to family 61 glucanase	11.29
3289_g (PIIN_05863)	Endo-1,4-beta-xylanase	10.75
7464_g (PIIN_04708)	Alpha-L-arabinofuranosidase	10.14
5322_g (PIIN_05889)	Endo-1,4-beta-xylanase	10.14
1898_g (PIIN_08141)	Glutathione S-transferase	9.1
5131_g (PIIN_02752)	Cellulose 1,4-beta-cellobiosidase	8.93
3537_g (PIIN_10118)	Carboxylic ester hydrolase	8.83
6726_g (PIIN_08399)	Probable alpha-galactosidase B	8.29
4844_g (PIIN_06890)	Endo-1,4-beta-xylanase A	7.9
8585_g (PIIN_01553)	Probable beta-glucosidase	7.88
3597_g (PIIN_06117)	Related to endoglucanase B	7.78
5420_g (PIIN_07414)	Related to NACHT/WD40 domain-containing protein	7.64
1875_g (PIIN_06862)	Rhamnogalacturonan acetylesterase	7.62
6520_g (PIIN_06360)	Endo-1,4-beta-xylanase C	7.53
3290_g (PIIN_05862)	Probable endo-1,4-beta-xylanase A	7.42
3615_g (PIIN_11270)	Probable feruloyl esterase C	7.37
5971_g (PIIN_04536)	Probable gEgh 16 protein	7.3
8031_g (PIIN_01484)	Related to CEL1 protein precursor	7.2
6665_g (PIIN_03039)	Probable beta-glucoside glucohydrolase	7.15
720_g (PIIN_06594)	Cellulose 1,4-beta-cellobiosidase	7.08
3514_g (PIIN_09664)	Glucose oxidase	7.01
290_g (PIIN_04439)	Related to peroxisomal short-chain alcohol dehydrogenase	6.97
6967_g (PIIN_00353)	Exocellobiohydrolase 3	6.7
1893_g (PIIN_08147)	Probable glutathione S-transferase	6.57

DEGs are calculated as: colonized root vs. *Si* axenic culture exhibiting significant (padj. < 0.05) down- or up-regulation, log_2_ FC (fold change) during colonization

**Table 2:** Top 25 *Brachypodium distachyon* (*Bd*) differentially expressed genes (DEGs) during root colonization (4 DPI)

Gene	Description	log2FC
BdiBd21-3.2G0197800	MYB-related transcription factor	− 2.41
BdiBd21-3.5G0123400	ABA/WDS induced protein	− 2.14
BdiBd21-3.3G0280400	Putative glycosyltransferase family 28	− 2.06
BdiBd21-3.3G0558500	Putative steroid 17-alpha-monooxygenase	− 2.03
BdiBd21-3.3G0660200	AP2 domain-containing protein	− 2.02
BdiBd21-3.1G0813200	GRAS transcription factor	− 1.96
BdiBd21-3.3G0264400	Homologous to barley constans-like protein CO8	− 1.94
BdiBd21-3.4G0000100	Fantastic four meristem regulator FAF	− 1.84
BdiBd21-3.1G0416000	Hydrophobic Protein RCI2	− 1.69
BdiBd21-3.1G0002200	Ca2+-independent phospholipase A2	− 1.67
BdiBd21-3.1G0887100	Putative pseudo-response regulator 7	− 1.64
BdiBd21-3.4G0311800	Dirigent-like protein	− 1.6
BdiBd21-3.1G0815300	SPX domain-containing protein 3	− 1.43
BdiBd21-3.1G0972800	Cold regulated protein 27	− 1.42
BdiBd21-3.4G0303000	Putative protein kinase	− 1.32
BdiBd21-3.2G0034900	Putative sulfoquinovosyltransferase SQD2	− 1.21
BdiBd21-3.1G0281100	SPX – domain containing protein 3	− 1.23
BdiBd21-3.1G0554700	Anthranilate O-methyltransferase	− 1.18
BdiBd21-3.1G0584400	Peroxidase	− 1.17
BdiBd21-3.5G0205300	Putative calmodulin-dependent protein kinase	− 1.15
BdiBd21-3.2G0749200	Probable lipid transfer LTP2	− 1.13
BdiBd21-3.5G0303700	Wound-induced protein	− 1.1
BdiBd21-3.5G0024800	Heat shock protein 90-1	− 1.07
BdiBd21-3.1G0399200	bZIP transcription factor	− 1
BdiBd21-3.1G0557300	BURP domain protein	− 0.93
BdiBd21-3.3G0203000	Cupin-domain protein	6.13
BdiBd21-3.1G0469800	Glutathione S-Transferase	5.09
BdiBd21-3.4G0405200	Protein Hothead/ FAD binding	4.93
BdiBd21-3.3G0354800	Cytochrome P450 76C1	4.03
BdiBd21-3.3G0136300	Proprotein convertase subtilisin/kexin	3.87
BdiBd21-3.4G0068000	Pathogenesis-related protein Bet v I family	3.86
BdiBd21-3.1G0662500	LRR receptor-like serine/theronine protein kinase	3.57
BdiBd21-3.4G0556000	Alcohol dehydrogenase	3.39
BdiBd21-3.1G0772700	Pathogenesis-related protein 1 (PR1)	3.21
BdiBd21-3.4G0393500	Putative chalcone synthase	2.44
BdiBd21-3.3G0195800	Tryptophan decarboxylase	2.3
BdiBd21-3.4G0171000	Multicopper oxidase	2.28
BdiBd21-3.3G0639500	Glycosyl hydrolase protein/Chitinase-related	2.12
BdiBd21-3.1G0129100	Potato inhibitor I family	2.06
BdiBd21-3.2G0160100	Pipecolate/sarcosine oxidase	2.04
BdiBd21-3.4G0189100	Putative LRR protein kinase	1.99
BdiBd21-3.2G0418600	WRKY transcription factor	1.9
BdiBd21-3.4G0073800	Thaumatin family protein	1.8
BdiBd21-3.2G0545400	LRR protein	1.79
BdiBd21-3.4G0121800	Tryptophan biosynthesis protein	1.71
BdiBd21-3.4G0397700	Serine/threonine protein kinase	1.7
BdiBd21-3.4G0026800	Putative protein kinase	1.69
BdiBd21-3.2G0468100	Peroxidase	1.68
BdiBd21-3.2G0600500	Wall-associated receptor kinase	1.6
BdiBd21-3.2G0233800	PGP-like phosphoglycoprotein auxin transporter	1.58

DEGs are calculated as: colonized root vs. mock-inoculated root exhibiting significant (padj. < 0.05) down- or upregulation, log_2_ FC (fold change) during colonization

### Prediction of *Si* RNAi genes

Since RNAi-mediated gene silencing has been documented in most but not all fungi [42], we assessed whether the Si-2020 genome encodes RNAi-related proteins with conserved domain architecture and homology to RNAi components in the model filamentous fungus *Neurospora crassa* [43]. Genes encoding predicted DCLs (G4U2H0, G4TBW9) with typical domains (dsRNA-binding, RNase III and helicase, [44]), QDE2-like proteins with PIWI domains typical of AGOs (G4TEK0, G4TLO4, [45]), an AGO-like protein (G4T5G9), and RdRPs (G4TNU7, G4TQP0) were identified and were expressed in axenic culture and Bd21-3-associated *Si* samples (Additional file 2: Table S5). Thus, the *Si* genome is predicted to contain genes encoding critical components of the RNAi machinery. Based on these new data, and the earlier discovery that AGO and DCL families are expanded in the *Bd* genome [35], we decided to sequence the sRNAs of both organisms, in order to assess the role of RNAi-based regulation and communication in symbiosis.

### sRNA profiles undergo a substantial change at the onset of the *Si*-*Bd* symbiosis

To evaluate how the mutualistic interaction affects the sRNA profiles in the colonized root and respective *Si* and *Bd* controls, reads from Bd-C, Bd-Si, and Si-ax sRNA data sets were subjected to consecutive filtering steps (Additional file 2: Figure S6). This greatly reduced the number of raw reads to be analyzed and allowed us to distinguish between sRNAs with potential targets in the interacting organism (putative ck-sRNAs) and sRNAs with potential functions in the same organism (putative endogenous sRNAs) (Additional file 2: Table S6).

Analysis of sRNAs from the Bd-Si dataset revealed that the total number of putative ck-sRNAs exceeds that of endogenous sRNAs in both *Si* (786,732 vs. 261,478) and Bd21-3 (17 million vs. 1.6 million), but the converse is true for unique sRNAs (36,163 endogenous vs. 35,895 putative ck-sRNAs in *Si* and 483,352 endogenous vs. 286,198 putative ck-sRNAs in Bd21-3).

### Size distribution profiles of *Si* and Bd21-3 sRNAs

**Fig. 4:**
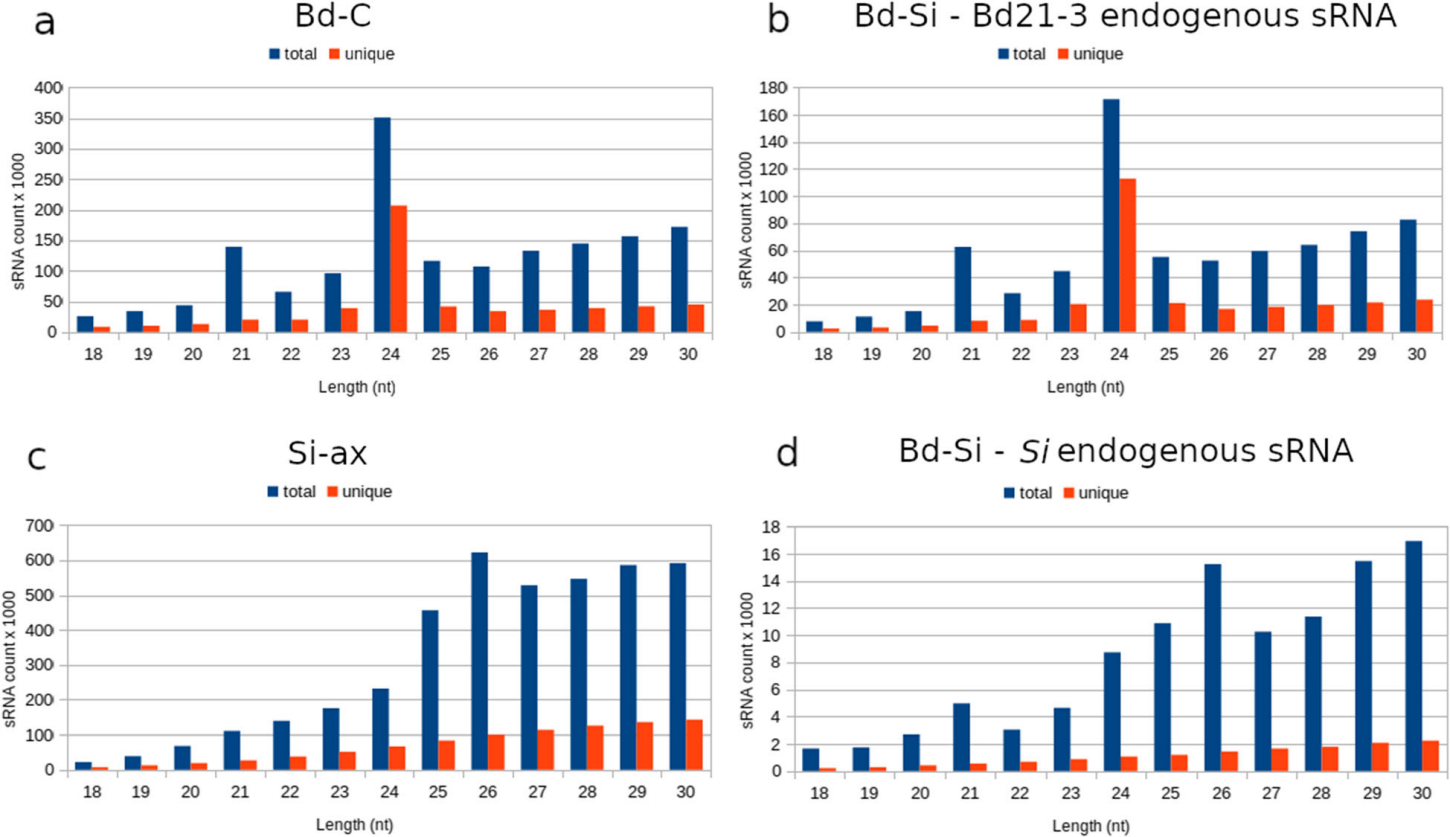
Size distribution of total and unique putative endogenous sRNAs. **a** Bd-C (mock-treated), **b** Bd-Si (colonized root), **c** Si-ax (axenic mycelium), and **d** Bd-Si (colonized root). All datasets represent three biological replicates and corresponding two technical replicates, merged together. sRNA length is displayed on the *X*-axis (nt) and number of total/unique sRNA counts on the *Y*-axis (× 10^3^)

Size distribution of sRNA reads from Bd21-3 and *Si* during colonization, and the respective controls was then assessed. For putative endogenous Bd21-3 sRNAs, peaks at 21 and 24 nt were identified, with the 24 nt sRNAs exhibiting greater diversity than those of 21 nt (Fig. 4a, b). These sizes are consistent with the expected peaks of RNAi-associated sRNAs in plants [46]. For putative endogenous *Si* sRNAs, a bimodal size distribution pattern was observed in the total fractions, with the first peak at 26 nt and second at 29–30 nt (Fig. 4c, d). A smaller peak of 21 nt long molecules was observed in the Bd-Si but not Si-ax samples, indicating that colonization affects the relative size distribution of *Si* sRNAs. Since previously identified ck-sRNAs range from 20 to 24 nt [12, 13], the size distribution of putative ck-sRNAs and corresponding reads in the control samples was assessed over a narrower window. Contrary to endogenous sRNAs, ck-sRNAs displayed no prominent peaks in the 20–24 nt window (Additional file 2: Figure S7).

Before sRNAs can guide RNAi-mediated gene silencing, they must be loaded onto AGO proteins and assembled into a RISC. Previously, Arabidopsis AGO proteins were shown to preferentially recruit sRNAs with specific 5′ termini [47]. Hence, we analyzed the 5′ terminal nt composition of Bd-C, Si-ax, and Bd-Si sRNAs. For unique putative endogenous and ck-sRNAs, the 5′ nt composition was relatively consistent except for the 24 nt Bd21-3 sRNAs, which exhibited a strong bias towards 5′ A (Additional file 2: Figure S8, Figure S9). The total sRNA fractions exhibited somewhat greater variability in 5′ nt composition. Of the total Bd21-3 endogenous sRNAs, 24 nt molecules from colonized and non-colonized tissue showed a strong bias towards 5′ A, while 21 nt molecules were biased towards a terminal U (Additional file 2: Figure S10), and 20 nt ck-sRNAs had a higher percentage of 5′ Cs (Additional file 2: Figure S11). Of the total endogenous *Si* sRNAs, those from colonized samples generally had a stronger bias towards 5′ A than sRNA reads from Si-ax, especially at 26 nt and 21 nt (Additional file 2: Figure S10). A slightly higher percentage of 5′ As also was detected in total putative ck-sRNAs of 21 nt (Additional file 2: Figure S11).

### Differentially expressed *Si* and Bd21-3 sRNAs

**Fig. 5:**
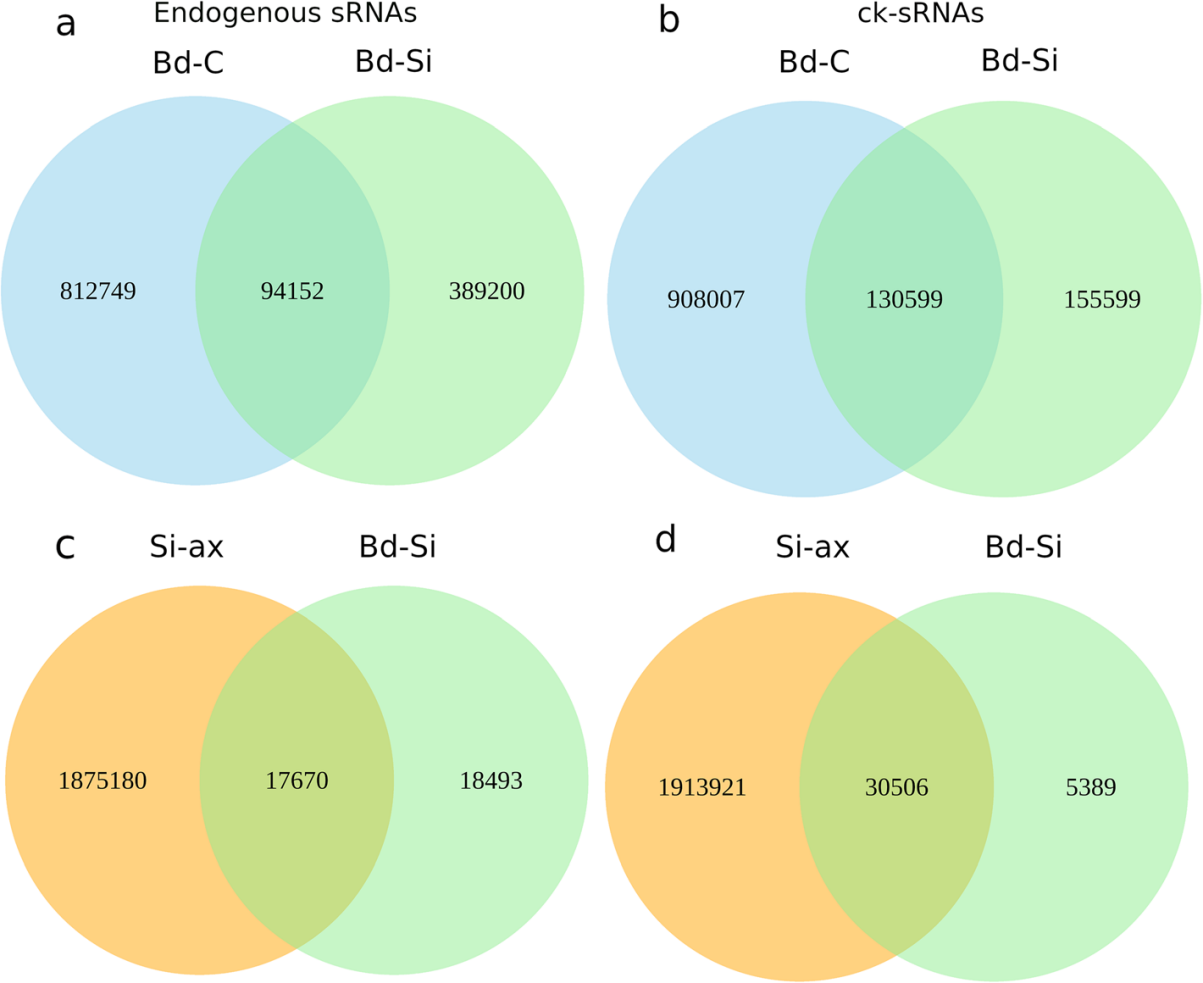
Venn diagrams showing the sample-exclusive or communal presence of unique putative endogenous or ck-sRNAs. **a** putative endogenous sRNAs in Bd-C (mock-treated) vs. Bd-Si (colonized root), **b** sRNA reads in Bd-C vs. putative ck-sRNAs in Bd-Si, **c** putative endogenous sRNAs in Si-ax (axenic mycelium) vs. Bd-Si (colonized root), and **d** sRNA reads in Si-ax vs. putative ck-RNAs in Bd-Si

Analysis of unique plant sRNAs in Bd-C vs. Bd-Si revealed that 63% of the putative endogenous sRNAs were exclusively present in Bd-C, 30% were exclusively in Bd-Si and 7% were present in both (Fig. 5). For the reads from the putative ck-sRNA pipeline, 76% of the reads were exclusively present in Bd-C, 13% were exclusive to Bd-Si, and 11% were found in both. Comparison between the unique fungal sRNAs in Si-ax and Bd-Si indicated that 98.1% of the putative endogenous sRNAs were exclusively present in axenic culture, 0.98% were exclusively found in Bd-Si, and 0.92% were in both. Similarly, from the putative ck-sRNA pipeline, 98.2% of the sRNA reads were exclusive to the Si-ax sample, 0.3 % were exclusive to Bd-Si, and 1.5% were present in both. Considering only the *Si* sRNAs in the Bd-Si sample, 51.1% of the putative endogenous ones and 15% of the putative ck-sRNAs are exclusively present in the colonized sample. Among Bd21-3 sRNAs in the Bd-Si sample, there are 80.5% putative endogenous sRNAs and 54.3% ck-sRNAs exclusive for the colonized root. These data show that colonization induces many novel putative endogenous and ck-sRNAs in Bd21-3, and a smaller amount in *Si*, due to fungal quantity in the colonized roots*.*

### Identification of Bd21-3 miRNAs during the *Bd-Si* interaction

Using the ShortStack analysis tool, we identified Bd21-3 loci that correspond to putative endogenous sRNAs expressed during *Si* colonization. The DicerCall function indicated loci whose predominant sRNAs are 20–24 nt. Comparison of these sRNAs with miRBase identified 16 sRNA-generating loci that correlate to known miRNAs (Table 3). These miRNAs belong to highly conserved plant miRNA families that regulate growth and development [33, 34]. We conducted the same analysis with *Si* sRNAs, but no predicted miRNA-like RNAs were identified in the colonized sample, possibly due to a lack of data about the fungal sRNA-generating loci.

**Table 3:** Predicted miRNA-generating loci identified in Bd21-3 roots colonized by *Serendipita indica*

Locus	Predominant sRNA	Known miRNA
Bd1:31073434-31073613	UCGGACCAGGCUUCAUUCCCC	bdi-MIR166b
Bd2:10327043-10327213	CUGCACUGCCUCUUCCCUGGC	bdi-MIR408
Bd2:3991996-3992115	UGACAGAAGAGAGUGAGCAC	bdi-MIR156e
Bd2:3992232-3992320	UGACAGAAGAGAGUGAGCAC	bdi-MIR156f
Bd2:3992444-3992557	UUGACAGAAGAGAGUGAGCAC	bdi-MIR156g
Bd2:5570263-5570488	CUUGGAUUGAAGGGAGCUCU	bdi-MIR159a
Bd3:1968409-1968509	UCGCUUGGUGCAGAUCGGGAC	bdi-MIR168
Bd3:33173606-33173746	UCGGACCAGGCUUCAUUCCCC	bdi-MIR166c
Bd3:39150748-39150893	GCUCACUUCUCUCUCUGUCACC	bdi-MIR156b
Bd3:4482899-4483000	UUGACAGAAGAGAGUGAGCAC	bdi-MIR156c
Bd3:44836298-44836372	AGAAGAGAGAGAGUACAGCCU	bdi-MIR529
Bd3:7316295-7316393	GGGCAACUCCUCCGUUGGCAGA	bdi-MIR399d
Bd4:1654850-1654943	UGAAGCUGCCAGCAUGAUCUGA	bdi-MIR167e
Bd4:4893304-4893422	CGGAGGUCAGGAAUUCUACUGAUU	bdi-MIR9481b
Bd4:6029321-6029440	UCUCGGACCAGGCUUCAUUCC	bdi-MIR166f
Bd5:18466111-18466202	UGACAGAAGAGAGUGAGCAC	bdi-MIR156d

### In silico target prediction of putative *Si* and Bd21-3 ck-sRNAs

Since most examples of sRNA-based communication in plant-microbe interactions have the commonality of 21 nt long sRNAs that silence transcripts in the target organism [12, 13, 48], we predicted the targets of 21 nt colonization-induced *Si* and *Bd* putative ck-sRNAs and assessed their expression after colonization. Of 16,003 unique Bd21-3 targets predicted for 412 induced 21 nt *Si* sRNAs, 49 were confirmed as downregulated at 4DPI. This represents 15.4% of all significantly changed genes in Bd21-3 during *Si* colonization. Some 89% of these transcripts are predicted as targets of *Si* sRNAs that are expressed exclusively in colonized tissue or with log_2_FC > 1. A representative set of sRNA-mRNA duplexes, chosen based on target identity and expression of target and sRNA, indicates that putative ck-sRNA targets in Bd21-3 are associated with transcription factor families, signaling pathways, and basal plant defense (Table 4, Additional file 2: Table S7).

**Table 4:** Examples of deduced duplexes of *Serendipita indica* sRNAs and their downregulated targets in *Brachypodium distachyon*

sRNA name	sRNA Expression	Target transcript	Description	Transcript expression
*Si*sRNA 1	NA	BdiBd21-3.1G0887100.1	Homologous to Arabidopsis pseudo-response regulator 3 and 7	− 1.63
		BdiBd21-3.2G0440200.1	Serine-carboxypeptidase-like 26-related	− 0.81
*Si*sRNA 2	NA	BdiBd21-3.1G0399200.1	bZIP transcription factor	− 1.00
*Si*sRNA 3	NA	BdiBd21-3.2G0288400.1	LURP1-related	− 1.20
*Si*sRNA 4	NA	BdiBd21-3.1G0475100.1	Zinc-finger of the FCS-type	− 1.00
*Si*sRNA 5	0.13	BdiBd21-3.1G0047100.1	Nitrogen metabolic regulation protein NMR-related	− 0.78
*Si*sRNA 6	1.35	BdiBd21-3.3G0750900.1	Peroxygenase	− 0.77
*Si*sRNA 7	NA	BdiBd21-3.1G0917100.1	Enolase	− 0.52
*Si*sRNA 8	NA	BdiBd21-3.4G0303000.1	Protein kinase domain protein	− 1.31
*Si*sRNA 9	NA	BdiBd21-3.1G0759800.1	Carboxyl-esterase 15 related	− 0.86
*Si*sRNA 10	NA	BdiBd21-3.1G0813200.1	GRAS transcription factor	− 1.95
*Si*sRNA 11	NA	BdiBd21-3.1G1017200.1	Expansin-like related	− 0.84
*Si*sRNA 12	NA	BdiBd21-3.3G0134800.1	Copper transport protein ATOX1-related	− 1.11
*Si*sRNA 13	1.95	BdiBd21-3.2G0288400.1	LURP1-related	− 1.20
*Si*sRNA 14	NA	BdiBd21-3.1G0917100.1	Enolase	− 0.52
*Si*sRNA 15	NA	BdiBd21-3.4G0303000.1	Protein kinase domain protein	− 1.31
	NA	BdiBd21-3.1G0411900.1	Serine-type carboxypeptidase activity (Blast2GO)	− 0.82
*Si*sRNA 16	NA	BdiBd21-3.4G0507800.1	MYB transcrition factor	− 0.70
*Si*sRNA 17	NA	BdiBd21-3.1G0411900.1	Serine-type carboxypeptidase activity (Blast2GO)	− 0.82
*Si*sRNA 18	NA	BdiBd21-3.2G0269000.1	Mannose-binding lectin family	− 0.85
*Si*sRNA 19	NA	BdiBd21-3.3G0264400.1	Homologous to barley CONSTANS-like protein CO8	− 1.93
*Si*sRNA 20	NA	BdiBd21-3.5G0237900.1	Aquaporin transporter	− 1.02

sRNA expression was calculated as log_2_(colonized/control) from normalized reads, NA (not available) stands for sRNAs expressed exclusively in the colonized sample; transcript expression is indicated as the log_2_(colonized/control) FC

To assess whether Bd21-3 generates ck-sRNAs that potentially target *Si* genes, we searched for predicted targets of 329 Bd21-3 sRNAs (21 nt long) induced in *Si*-colonized roots. Of 3,019 predicted unique *Si* targets, 358 were confirmed as downregulated after colonization. This represents 12% of all significantly changed *Si* genes. 35% of the 358 *Si* transcripts are predicted to be targeted by Bd21-3 sRNAs exclusive to colonized tissue and an additional 27.6 % are targeted by sRNAs that are highly upregulated in colonized tissue (log_2_FC > 1). A set of sRNA-mRNA duplexes, selected with the same criteria as for the *Si* sRNA – Bd21-3 targets (Table 4), shows that predicted Bd21-3 ck-sRNAs have putative targets in *Si* which include proteins associated with nutrient acquisition, development of cell walls, hyphal networks, pathogenic fungal activities, fungal starvation, and signaling (Table 5, Additional file 2: Table S7). In order to confirm the expression of some of these sRNAs, we conducted stem-loop PCR and sRNA-specific sequencing on 10 *Si* and 10 Bd21-3-originating sRNAs from our Bd-Si sample and Table S7. All *Si* and all Bd21-3 sRNAs were amplified in the stem-loop PCR. To verify the nature of the amplification products, a subset of four *Si*sRNAs and three *Bd*sRNAs were cloned and sent for Sanger sequencing, confirming the expected sRNA sequences (Additional file 2: Figure S12, original gel pictures in Additional file 3 and Additional File 4, sequencing results in Additional file 2: Table S8). Thus, predicted targets of putative ck-sRNAs within this system imply another layer of expression control within the mutualistic interaction.

**Table 5:** Examples of deduced duplexes of *Brachypodium distachyon* sRNAs and their downregulated targets in *Serendipita indica*

sRNA	sRNA expression	Transcript	Description of target transcript	Transcript expression
*Bd*sRNA 1	NA	CCA68723	Related to phenylalanine ammonia-lyase	− 5.1
*Bd*sRNA 2	1.43	CCA69635	Probable acetyl-CoA synthetase	− 2.19
*Bd*sRNA 3	0.04	CCA72153	Putative mitochondrial carnitine O-acetyltransferase	− 2.94
*Bd*sRNA 4	0.58	CCA68099	Probable protein required for hyphal anastomosis HAM2	− 1.38
*Bd*sRNA 5	0.59	CCA69082	Related to serine/threonine-protein kinase	− 1.65
*Bd*sRNA 6	0.039	CCA67801	Probable isocitrate lyase	− 2.34
*Bd*sRNA 7	4.37	CCA68918	Probable ADH1-alcohol dehydrogenase I	− 4.42
*Bd*sRNA 8	NA	CCA77975	Related to peroxisomal membrane protein 4	− 2.39
		CCA68099	Probable protein required for hyphal anastomosis HAM2	− 1.38
*Bd*sRNA 9	0.029	CCA73455	Related to phosphoprotein phosphatase 2C	− 0.89
*Bd*sRNA 10	1.39	CCA72944	Protein TOXD	− 2.73
		CCA72668	Hypothetical protein	− 1.68
		CCA74115	Probable nucleolar rRNA processing protein GAR1	− 0.93
*Bd*sRNA 11	0.19	CCA77931	Related to iron transport protein	− 2.4
*Bd*sRNA 12	0.48	CCA68412	Related to ECM32-DNA dependent ATPase/DNA helicase B	− 1.96
*Bd*sRNA 13	1.06	CCA75416	Related to estradiol 17 beta-dehydrogenase 4	− 2.12
*Bd*sRNA 14	NA	CCA73650	Related to chitinase	− 1.37
*Bd*sRNA 15	0.49	CCA77900	Related to LSB5-possible role in the regulation of actin cytoskeletal organization	− 1.27
*Bd*sRNA 16	0.62	CCA69912	Related to acyl-CoA dehydrogenase	− 2.28
*Bd*sRNA 17	0.88	CCA70015	Related to CAT1	− 1.84
*Bd*sRNA 18	NA	CCA68373	Probable subtilisin-like serine protease	− 0.91
*Bd*sRNA 19	1.38	CCA72980	Hypothetical protein	− 2.59
		CCA67021	Probable VID27-involved in vacuole import and degradation	− 0.93
*Bd*sRNA 20	0.1	CCA73174	Related to ECM4-involved in cell wall biogenesis and architecture	− 2.48

sRNA expression was calculated as log_2_(colonized/control) from normalized reads, NA stands for sRNAs expressed exclusively in the colonized sample; transcript expression is indicated as the log_2_(colonized/control) FC

## Discussion

We established and studied the interaction between *Brachypodium distachyon*—a model Pooideae plant with shared synteny to major cereal crops—and *Serendipita indica*—a beneficial endophyte with an exceptionally large host range. This particular combination of traits adorning the *Bd-Si* interaction has great translational value towards filling in the gaps in knowledge about plant symbioses, especially their transcriptomic and sRNA expression profiles and the significance of RNAi. We show that *Si* colonizes *Bd*, resulting in shoot growth promotion, earlier flowering, and improved grain development. In comparison, earlier studies characterizing the interaction between *Si* and barley have demonstrated that fungal hyphae establish an interface with the root cell plasma membrane at an early colonization stage, followed by expansion of an extracellular hyphal network, intercellular growth, and intracellular penetration of cortical and rhizodermal cells [26]. Around 3 to 5 DPI, *Si* starts the switch from a biotrophic to a saprophytic lifestyle [26, 27]. Although this change involves intracellular hyphae extensively colonizing dead host cells and gradual digestion of cortical cell walls, the plant still benefits from the fungal presence. Consistent with these findings, our microscopic analyses confirmed proliferation of *Si* chlamydospores and both inter- and intracellular colonization of Bd21-3 cells in the root differentiation zone from 4 to 14 DPI. Detection of proliferating hyphae that were not wrapped in plasma membrane further suggests that *Si* is colonizing dead surface plant root cells at 4 DPI [25, 26].

### Transcriptional changes detected during the *Bd-Si* interaction

To investigate colonization of Bd21-3 by *Si*, we analyzed *Si* DEGs in colonized vs. axenic mycelium samples. Gene ontology analysis indicated enrichment in genes involved in various metabolic and catalytic processes. DEGs with the greatest changes in expression play roles in plant cell wall degradation, carbohydrate metabolism and nutrient acquisition. These changes in nutritional reprogramming are to be expected, considering the different nutritional content that *Si* has accessible in planta vs. axenic CM plates and a more detailed look into the roles of the changed genes unveils a typical switch of fungal lifestyle. Examples of upregulated *Si* genes involved in cell wall degradation include a probable *Pectate lyase*, *Endo-1,4-beta-xylanases*, *Cellulose 1,4-beta-cellobiosidase*, and *Rhamnogalacturonan acetylesterase*. The genes encoding these hydrolytic enzymes, which have undergone expansion in the *Si* genome [49], are similar to those upregulated in *Si* during saprophytic growth on autoclaved barley roots at 3 DPI and 5 DPI [22]. *PiAMT1*, encoding a high affinity ammonium transporter also was upregulated (logFC = 3.35; padj < 0.0001). Other upregulated genes encode enzymes involved in carbohydrate metabolism, including probable glucosidases, glucanase, and galactosidase. These proteins may modulate glucose concentration, which then regulates expression of some cell-wall degrading enzymes [22, 50]. Some of the 174 putative effector protein-encoding genes also are differentially expressed during *Si* colonization of barley and Arabidopsis [22, 27]. Six of these proteins (Additional file 2: Table S4) contain the *Si*-specific DELD domain, which suggests that *Si* utilizes a common protein effector arsenal to colonize various hosts. Considering highly downregulated *Si* DEGs, several encoded proteins are associated with adaption to nutrient availability (*Accumulation of dyads protein 2*, *ADY2*; *Succinate-fumarate transporter*) and nutrient acquisition (*Acyl-CoA dehydrogenase*; *Carnitine acetyltransferase*, *CRAT*; *Phenylalanine ammonia-lyase*, *PAL* [51, 52];). Their reduced expression suggests ample nutrient availability at 4 DPI. Given the similarities in the *Si* transcriptome during colonization of *Bd* and barley, we propose that these fungal-plant interactions follow a pattern, and that by 4 DPI, a network of plant-endophyte communication cues has initiated a tightly controlled transcriptional program, leading to a shift from biotrophic to saprophytic growth.

Roots of Bd21-3 plants also displayed substantial transcriptional reprogramming following *Si* colonization. Gene ontology term analysis indicated enrichment in genes involved in catalytic and oxidoreduction-associated processes. Bd21-3 DEGs exhibiting the greatest changes in expression between colonized and non-colonized plants are related to stress-response, defense, and plant development. Of the downregulated Bd21-3 genes, several encode proteins commonly associated with stress responses, including a peroxidase, a wound-induced protein*,* and a putative protein kinase*.* Additionally, members of the *Heat-shock protein* gene family [53] are commonly induced in *Bd* during abiotic stress and members of the *Abscisic acid/water deficit stress (ABA/WDS)-induced protein* and the *Rare cold inducible (RCI2)* gene families enhance abiotic stress tolerance in various plant species [54, 55]. Circadian clock and flowering regulation genes such as *Pseudo-response regulator 7* (*PRR7*), *Cold regulated protein 27* and *Constans-like protein* (*CO8*), also are downregulated during *Si* colonization. While members of the PRR and CO protein families work together to control flowering time [56, 57], any influence on early flowering in *Si*-colonized Bd21-3 is unclear. Circadian clock-associated genes also regulate lateral root development in Arabidopsis [58]; whether *Si*-induced changes in their expression influence root growth is unknown. Other downregulated development-associated DEGs include *Fantastic four meristem regulator* (*FAF*), which regulates shoot and root development [59], and putative *Sulfoquinovosyltransferase* (*SQD2*), which modulates seed setting and tiller development in rice [60]. Finally, several downregulated DEGs encode transcription factors, including MYB-related, GRAS, and bZIP.

In comparison, many of the upregulated Bd21-3 DEGs are associated with immune responses. Examples include genes encoding leucine-rich repeat (LRR) protein, a WRKY transcription factor, and thaumatin family protein. Increased expression of the defense gene *Pathogenesis-related protein 1* (*PR1*) was similarly and transiently reported in *Si*-colonized Arabidopsis roots [61]. Upregulation of G*lutathione S-transferase* (*GST*) is consistent with the increased antioxidant capacity of *Si*-colonized plants, which provides protection against attack by necrotrophic pathogens [21, 62]. The upregulation of genes in other hormonal networks (*PGP-like Phosphoglycoprotein auxin transporter*) and redox processes (*Multicopper oxidase*) further suggests that *Si* colonization affects a range of signaling pathways.

### *Bd* miRNAs detected in the Bd-Si sample

The role of miRNAs as regulators of gene expression in the Sebacinalean symbiosis is largely unexplored. One report showed that *Si* induces growth promotion-associated miRNAs in *Oncidium* orchid roots [63]. Analysis of putative endogenous Bd21-3 sRNAs expressed during *Si* colonization identified 16 miRNAs. Some of them have known targets in transcription factors associated with plant growth and development. For example, the bdi-MIR166 family targets mRNAs encoding *Homeobox domain-leucine zipper* transcription factors [64]. In Arabidopsis, repression of these transcription factors by the miR165/166 family modulates root growth, maintenance of the shoot apical meristem, and development of leaf polarity [65]. Plant-specific transcription factors encoded by *Squamosa promoter-binding protein-like* (*SPL*) genes are the presumed targets of bdi-MIR156 and bdi-MIR529 [66]. In Arabidopsis, miR156-mediated downregulation of SPLs modulates developmental timing, lateral root development, branching, and leaf morphology [65]. Members of the *MYB* superfamily of transcription factors, which regulate many aspects of development, are the predicted targets of bdi-MIR159 [34]. Interestingly, miRNAs belonging to the miR159 and miR166 families in cotton are known ck-sRNAs that target virulence genes in *Verticillium dahliae* [13].

Other miRNAs identified in Bd21-3 include bdi-MIR168, predicted to target *AGO1* [64], and two miRNAs that regulate nutrition: bdi-MIR399, which is upregulated in *Bd* by phosphate starvation [64, 67], whereas bdi-MIR408 influences copper levels [34, 68]. Additionally, bdi-MIR408 (*Bd*sRNA 10) has predicted ck targets in three *Si* transcripts: *CCA72944, CCA72668*, and *CCA74115*. Since various targets were predicted for bdi-MIR167 [34, 68] and no target was predicted for bdi-MIR9481, their endogenous functions in *Bd* are unclear. Interestingly, the miRNA families identified in our analysis, except bdi-MIR9481, also were detected in *Si*-colonized *Oncidium* [63]. Thus, this group of miRNAs may play an important role in reprogramming plant cells during Sebacinalean symbiosis establishment.

### Putative *Si* and Bd21-3 ck-sRNAs and their predicted targets

To date, cross-kingdom RNAi has been demonstrated in pathogenic plant-fungal interactions [12, 13, 69], and while there are promising indications for its presence during plant-mycorrhiza interactions [36], whether it occurs in *Si*-plant associations is unknown. To investigate this possibility, we predicted targets for 21 nt putative ck-sRNAs from *Si* and Bd21-3 and confirmed their downregulation during colonization. This analysis uncovered 358 downregulated *Si* transcripts that are the predicted targets of 228 unique Bd21-3 sRNAs. Cross-kingdom RNAi-mediated downregulation of these targets might allow Bd21-3 to modulate *Si* growth during colonization. For example, *PAL*, *Acetyl-CoA synthetase,* C*arnitine acetyl transferase*, *Isocitrate lyase*, and *Acyl-CoA dehydrogenase*, which are targeted by *Bd*sRNA 1, *Bd*sRNA 2, *Bd*sRNA 3, *Bd*sRNA 6, and *Bd*sRNA 16 (Table 5), are involved in fungal nutrient acquisition [22, 55, 70, 71]. Genes with important homologs in pathogenic fungi also are predicted targets, including S*ubtilisin-like serine protease* (*Bd*sRNA 18) [72], *Alcohol dehydrogenase 1* (*Bd*sRNA 7) [73], and *Phosphoprotein phosphatase 2C* (*Bd*sRNA 9) [74]. Targeting of *Hyphal anastomosis-2* (*HAM-2*) by *Bd*sRNA 8 and *Bd*sRNA 4 may provide another mechanism for controlling fungal growth, as HAM-2 is required for hyphal fusion in *N. crassa* [75]. Similarly, targeting of *Chitinase* (*Bd*sRNA 14) may help control *Si* growth.

Concurrently, we identified 49 downregulated Bd21-3 mRNAs that are the predicted targets of 63 unique *Si*-generated ck-sRNAs. Downregulation of these target genes via cross-kingdom RNAi might facilitate *Si* growth during colonization. For example, *Mannose-binding lectin* (targeted by *Sis*RNA 18) belongs to a family of defense-related genes whose products trigger immune responses following pathogen recognition [76]. *Si*sRNA 8 and *Si*sRNA 15 target a protein kinase domain/LRR gene (*BdiBd21-3.4G0303000.1*) that may belong to the LRR receptor kinase family, which regulates defense and developmental-related processes [77]. Transcripts encoding serine-carboxypeptidase-like (SCPL) proteins *BdiBd21-3.2G0440200.1* and *BdiBd21-3.1G0411900.1* (targeted by *Sis*RNA 1 and *Sis*RNA 15) are associated with defense against (a)biotic stresses in monocots [78]. Members of various transcription factors families also were identified as predicted targets (*MYB* by *Si*sRNA 16, *bZIP* by *Si*sRNA 2, and *GRAS* by *Si*sRNA 10). These families are associated with (a)biotic stress responses, as well as plant growth and development [79–81]. Lastly, transcripts for proteins involved in circadian clock and flowering regulation (*BdiBd21-3.1G0887100.1* and *BdiBd21-3.3G0264400.1* [56];) are the presumed targets of *Si*sRNA 1 and *S*isRNA 19. Together, these findings suggest that *Si*-derived ck-sRNAs may promote fungal colonization by targeting signaling processes associated with plant development and responses to (a)biotic stresses.

In combination with earlier studies on *Bd* RNAi proteins [35] and *Bd* interaction with the pathogen *Magnaporthe oryzae* [82], the in silico analyses presented here suggest that *Si* and *Bd* contain functional RNAi components and that both organisms generate ck-sRNAs, which potentially modulate this mutualistic interaction. However, further studies are necessary to validate cross-kingdom RNAi in a Sebacinalean symbiosis. Namely, degradome analysis is needed to confirm target degradation and evidence that Bd21-3 and *Si* AGOs associate with sRNAs expressed by the interacting organism is necessary for confirmation of cross-kingdom RNAi.

## Conclusions

We report that Bd21-3 and *Si* form a mutualistic symbiosis with a promoting effect on plant yield and development, accompanied by changes in gene expression in both organisms, including putative protein *Si* effectors and RNAi-related genes. sRNA profiles of both organisms also changed, indicating that this model system will provide important insights into the multiple layers of regulation and interaction between beneficial fungi and cereal hosts. Within the broader scope of plant-mutualist interactions, we show that detection of putative RNAi-involved sRNAs in an interaction highly benefits from simultaneous transcriptome analysis and indicate an involvement of sRNA-based regulation in defense responses, nutritional reprogramming, and colonization maintenance. Alongside other experimental approaches in plant-microbe interactions (eg. sRNA uptake studies [83]), developing a deeper understanding of the communication mechanisms that modulate mutualistic interactions is highly relevant for establishing robust growth promotion and protection strategies in crops.

## Methods

### *Bd* and *Si* cultivation and inoculation

The seeds of *Brachypodium distachyon* (*Bd*) line Bd21-3 (gift from R. Sibout, INRA Versailles, France) were surface sterilized (3% active chlorine, sodium hypochlorite solution) for 15 min, washed three times, and placed on half-strength MS [84] medium in dark at 4 °C for 2 days and then 7 days at 24 °C and 16 h light/8 h dark cycle (47 μmol m^− 2^ s^− 1^ photon flux density). *Serendipita indica* (*Si*) (IPAZ-11827, Institute of Phytopathology, Giessen, Germany) was grown on complete media plates (CM [85]) at 23 °C in dark for 4–5 weeks.

For inoculation, *Si* mycelium was collected in 0.002% aqueous Tween 20 solution, filtered (Miracloth, Calbiochem), and pelleted by centrifugation (10 min/4000 rpm/20 °C) twice. Chlamydospore concentration of 5 × 10^5^ conidia ml^−1^ in 0.002% Tween 20 solution was used to inoculate 7-day-old plant seedlings for 2–3 h. Control plants were mock treated with the 0.002% Tween 20 solution for the same time. Grain yield analyses were done on mature plants grown on soil (F-E type LD 80, Fruhstorfer Erde, Germany) under 16 h light (160 μmol m^−2^ s^−1^, 22 °C) and 8 h dark (18 °C) conditions at 60% relative humidity for 1 month, and then greenhouse conditions until seed maturity. Number of spikelets was assessed after 2 months. Shoot biomass was assessed 3 weeks after inoculation of seedlings grown on a mixture (2:1, v/v) of vermiculite (Deutsche Vermiculite GmbH) and oil dri (oil binder Typ III R Coarse grain, Damolin, Mettmann, Germany) under comparable conditions as for grain yield, and fertilized every 3 days with an aqueous solution of Wuxal Super NPK-8/8/6 (1:10^3^ v/v; Haug, Düsseldorf, Germany). Samples for RNA-seq, RT-qPCR, stem-loop PCR, and microscopy were also grown under these conditions. To assess growth promotion in *Si* inoculated *Bd* relative to the control, we used the pairwise *t* test or the Mann-Whitney-Wilcoxon test on each of the three repetitions of experiments, after checking for normality and homogenous variances. Benjamini-Hochberg correction for multiple testing was used to correct the *p* values and the significance asterisks were assigned to the average p-value as follows: * for *p* ≤ 0.05, ** for *p* ≤ 0.001, and *** for *p* ≤ 0.0001.

### Microscopy

Following *Si* inoculation, one-week-old seedlings were grown on plastic mesh (~ 90 μm) placed over half-strength MS medium or on vermiculite/oil dri prior to assessing root colonization. *Si* was visualized with the chitin-specific dye WGA-AF 488 (wheat germ agglutinin; Molecular Probes, Karlsruhe, Germany), as described in Deshmukh *et al*. (2006) [26], with boiling in KOH (10%) for 30 s, prior to incubation in phosphate-buffered saline (PBS, pH 7.4). Root cells were visualized by incubating with propidium iodide (10 μg ml^−1^) for 10 min and washing with sterile water. Confocal laser scanning microscopy was done (TCS SP8 microscope, Leica, Bensheim, Germany) and the Leica LAS X software was utilized for visualization and maximum (*z*-stack) projections.

### Resequencing, assembly, and annotation of the *Si* genome

The MasterPure Yeast DNA Purification Kit (Epicentre, Illumina) was used to extract genomic DNA from 4-week-old axenic *Si* cultures. The *Si* genome was resequenced, assembled [86], and annotated as described [87], whereby a MinION sequencing library was prepared using the Nanopore Rapid DNA Sequencing kit. Sequencing was performed on an Oxford Nanopore MinION Mk1b sequencer using a R9.5 flow cell. Additionally, sequencing of an Illumina Nextra XT library was performed on the MiSeq platform (Illumina; 2 × 300 bp paired-end sequencing, v3 chemistry). Adapters and low-quality reads were removed by an in-house software pipeline prior to polishing [88]. MinKNOW (v1.13.1, Oxford Nanopore Technologies) was used to control the run with the 48 h sequencing run protocol, and base calling was performed offline using albacore (v2.3.1, Oxford Nanopore Technologies). The assembly was performed using Canu v1.6 ([89], default settings). The resulting contigs were polished with Illumina short read data using Pilon [90] for eight iterative cycles. BWA-MEM [91] was used for read mapping in the first four iterations and Bowtie2 v2.3.2 [92] in the second set. Gene prediction was performed with GeneMark-ES 4.3.3. ([38], default settings). Predicted genes were functionally annotated using a modified version of the genome annotation platform GenDB 2.0 [39] for eukaryotic genomes [40]. RNAi-associated proteins were predicted by searching the proteome [22] for typical domain structure and highest homology to *Neurospora crassa* RNAi proteins (NC12 genome assembly [93]). A modified version of the pipeline from Rafiqi et al. (2013) [41] was used to predict protein effectors. After identifying proteins with signal peptides (signalp-4.1 [94]), those predicted as transmembrane helix proteins (tmhmm [95]), mitochondrial proteins (target-1.1 [96]), and cell wall hydrolysis-associated proteins were removed. For comparative analysis of the *Si* (Si-2020 and DSM11827 ASM31354 v.1 [22]), *Serendipita vermifera* [97] and *Laccaria bicolor* [98] genomes, software platform EDGAR 2.3 [99] was used.

### RNA extraction, library preparation, and mRNA/sRNA sequencing

Roots inoculated with *Si* (Bd-Si) or mock-inoculated (Bd-C), as described above, were grown for 4 days and pooled (three roots per sample). *Si* mycelium and spores were collected from 4-week-old axenic cultures grown on CM medium. All samples in triplicates were shock frozen, stored at − 80 °C, and ground in liquid N_2_. Total RNA was isolated using the ZymoBIOMICS RNA Mini Kit (Zymo Research, USA), quantified with DropSense16/Xpose (BIOKÉ, Netherlands), and analyzed with an Agilent 2100 Bioanalyzer Nano Chip (Agilent, Germany). RNA Clean and Concentrator 25 and 5 kits (Zymo Research) were utilized to separate total RNA into fractions: 17–200 nt and > 200 nt. 1.5 μg of the larger fractions were processed for mRNA library preparation (TruSeq Stranded mRNA protocol, Illumina, USA). Fragment Analyzer Automated CE System (Advanced Analytical Technologies, Austria) determined the quality of the generated polyA mRNA libraries. Quantity and quality of the smaller RNA fractions were assessed with the Qubit fluorometer (Invitrogen, Germany) and Agilent 2100 Bioanalyzer Pico Chip. sRNA library preparation was done with 50 ng of RNA (TruSeq Small RNA Library Prep, Illumina) and size selection with the BluePippin (Sage Science, USA) for fragments between 140 and 160 nt (15–35 nt without adapters) applied. Sequencing was accomplished on the Illumina HiSeq 1500.

### Transcriptome analysis

Raw reads from mRNA sequencing [100] were submitted to quality check using FastQC [101] and aligned to the Bd21-3 v1.1 (DOE-JGI, http://phytozome.jgi.doe.gov/ [102]) or resequenced *Si* (Si-2020) genomes with HISAT2 [103]. An intron length of 20–2000 nt was allowed for *Si* [104] and 20–10,000 nt for Bd21-3 [105]. The reads were counted using HTSeq-count [106], differential gene expression was performed with DESeq2 [107], and gene enrichment analysis with AgriGO v.2 [108], with reference Bd21 setting for Bd21-3 (Bd 21 synonyms) and a customized background for *Si*. Volcano plots were generated using plotly [109] and ggplot2 [110] R [111] libraries. Gene descriptions were obtained from the organism annotations or Blast2GO [112].

### sRNA analysis and prediction of putative endogenous and ck-sRNAs

Raw reads from sRNA sequencing [113] were submitted to FastQC [101] and adapter trimming [114]. Bowtie [115] was used for alignment as detailed in Additional file 2: Fig. S6. The resequenced *Si* genome was used for alignments of fungal origin. tRNA/rRNA sequences were downloaded from RNAcentral ([116], EMBL-EBI). Putative endogenous sRNA reads were submitted to ShortStack [117]. For filtering putative ck-sRNAs, a previously established pipeline [118] was utilized. Reads were normalized to the total number of mapped reads for a single genome and reads per million (RPM) and log_2_ (colonized/mock-treated) values calculated. Thus, a sRNA read was selected as a putative ck-sRNA if it was present exclusively or at a higher quantity (i.e., induced) in the colonized vs. control sample. Putative ck-sRNAs were submitted to psRNAtarget [119]. Since the separation of sRNA and mRNA fractions from each biological sample was facilitated, we checked for downregulation of mRNAs corresponding to predicted sRNA target genes within the DEGs. Transcriptomes used for these predictions were Bd 21-3 v1.1 (DOE-JGI, http://phytozome.jgi.doe.gov/ [102]) and *Si* DSM11827 ASM31354 v.1 [22]. Venn diagrams were generated using the VennDiagram R package [120].

### Quantitative real-time PCR and stem-loop PCR for validation of sequencing results

To validate gene expression detected in the sequencing, we used quantitative real-time PCR (qRT-PCR). RNA extraction from mock treated and *Si* inoculated Bd21-3 roots, as well as *Si* axenic cultures, under the same conditions as explained above for the sequencing, was done with TRIzol (Thermo Fisher Scientific, Waltham, MA, USA), cDNA synthesized using qScriptTM cDNA kit (Quantabio, Beverly, MA, USA) and 10 ng of cDNA used as template in the QuantStudio 5 Real-Time PCR system (Applied Biosystems), with SYBR® green JumpStart Taq ReadyMix (Sigma-Aldrich, St. Louis, MO, USA). Each sample had three technical replicates. Primers used for these amplifications are listed in Table S9 (Additional file 2: Table S9). Transcript levels were calculated using the 2–ΔΔCt method [121], relatively to *BdUbi4-3* for Bd21-3 and *Si ITS* sequence for *Si*.

For the identification of sRNAs in the interaction of *Si* with Bd21-3 stem-loop RT-PCR was employed [122]. cDNA was synthesized from DNase I-treated total RNA extracted from *Si* axenic culture or inoculated Bd21-3 roots. The folding of the hairpin primer was performed according to Kramer (2011) [123]. For each stem-loop reaction, six hairpin primers were multiplexed in a 20-μL reaction using the Revertaid RT enzyme according to the manufacturer’s instructions (Thermo Scientific). For primer annealing, the reaction was incubated for 30 min at 16 °C followed by an extension step at 42 °C for 30 min. The reaction was stopped at 85 °C for 5 min. cDNA was stored at − 80 °C until further use. Endpoint PCR was performed using an universal stem-loop primer and specific sRNA primer (Additional file 2: Table S10) under the following conditions: initial denaturation at 95 °C for 5 min followed by 35 cycles: 95 °C for 30 s, primer annealing at 60 °C for 30 s, and extension at 72 °C for 30 s. PCR products were separated by gel electrophoresis on a 2% (*w*/*v*) agarose gel. To obtain sequence information of the amplified sRNAs of the stem-loop reaction, PCR products were purified and cloned into the pGEM®-T Easy Vector Systems (Promega, Madison, WI, USA) following the manufacturer’s instructions. From each cloned sRNA, six colonies were further analyzed by Sanger sequencing using a M13 reverse primer.

## Supplementary Information

**Additional file 1.** Supporting individual data values displayed in Figure 1a-c and Additional File 2: Figure S5.

**Additional file 2: Fig. S1.***Serendipita indica* (*Si*) colonization has an effect on *Brachypodium distachyon* (*Bd*) root structure. **Fig. S2.** Progress of *Serendipita indica* (*Si*) spore proliferation during colonization of *Brachypodium distachyon* Bd21-3. **Fig. S3.** Comparison of the resequenced *Serendipita indica* (*Si*) genome to the 2011 assembly. **Fig.S4.** Comparison of the resequenced *Serendipita indica* (*Si*) genome to *Serendipita vermifera* and *Laccaria bicolor.* **Fig. S5**. qRT-PCR confirmation of DEGs identified during mRNA sequencing. **Fig. S6.** Filtering pipelines applied in the analysis. **Fig. S7.** Size distribution of total and unique putative ck (cross-kingdom) -sRNAs. **Fig. S8.** Percentage distribution of the 5′ terminal nucleotide in unique putative endogenous sRNAs. **Fig. S9.** Percentage distribution of the 5′ terminal nucleotide in unique putative ck-sRNAs. **Fig. S10.** Percentage distribution of the 5′ terminal nucleotide in total putative endogenous sRNAs. **Fig. S11.** Percentage distribution of the 5′ terminal nucleotide in total putative ck-sRNAs. **Fig. S12.** Stem-loop PCR (gel electrophoresis) of some *Si* and Bd21-3 sRNAs expressed in the Bd-Si sample. **Table S1.** Quantification of identified features in the resequenced genome of *Serendipita indica* (*Si*)*.* **Table S2.** Total reads from the Bd-C (mock-treated), Bd-Si (colonized root) and Si-ax (axenic culture) samples and their alignment rate (HISAT2) to the corresponding genomes. **Table S3.** Significant gene ontology terms of molecular function in the differentially expressed genes (DEGs) datasets. **Table S4.** Predicted protein effectors identified in the resequenced *Serendipita indica* (*Si*) genome. **Table S5.** Candidate RNAi machinery proteins predicted from the resequenced *Serendipita indica* (*Si*) genome. **Table S6.** Total and unique reads for filtered sRNAs from Bd-C (mock-treated), Bd-Si (colonized root) and Si-ax (axenic culture). **Table S7.** Sequences of putative sRNAs in Tables 4 and 5. **Table S8**. sRNA sequences after stem-loop PCR amplification. **Table S9.** Primers used for qRT-PCR (Figure S5). **Table S10.** Primers used for stem-loop PCR and sequencing (Figure S12, Table S8).

**Additional file 3.** Uncropped gel picture annotated in Additional file 2: Figure S12a.

**Additional file 4.** Uncropped gel picture annotated in Additional file 2: Figure S12b.

## Data Availability

All data generated or analyzed during this study are included in this published article, its supplementary information files (Additional files 1, 2, 3 and 4) and publicly available repositories. mRNA and sRNA-seq of *Brachypodium distachyon* roots inoculated with *Serendipita indica* datasets are available in the ArrayExpress database at EMBL-EBI (www.ebi.ac.uk/arrayexpress) under accession numbers E-MTAB-10649 and E-MTAB-10650, respectively. The genome assembly data for *Serendipita indica* resequencing study is available in the European Nucleotide Archive (ENA) at EMBL-EBI under accession numbers: assembly GCA_910890315 and study PRJEB45884 (https://www.ebi.ac.uk/ena/browser/view/PRJEB45884). Additional publicly available datasets have been accessed from the corresponding European Nucleotide Archive accessions and EnsemblFungi: *Serendipita indica* 2011 genome (GCA_000313545.1) [22], NC12 of *Neurospora crassa* (GCA_000182925.2) [93]. *Serendipita vermifera* subsp. bescii [97] has been accessed from JGI MycoCosm (https://mycocosm.jgi.doe.gov/Sebvebe1/Sebvebe1.home.html), as was the *Laccaria bicolor* genome ([98], https://mycocosm.jgi.doe.gov/Lacbi2/Lacbi2.home.html). The Bd21-3 v1.1 genome [102] was accessed under early access conditions and these sequence data were produced by the US Department of Energy Joint Genome Institute - DOE-JGI, http://phytozome.jgi.doe.gov/.

## Abbreviations

AGO: Argonaute
*Bd*: *Brachypodium distachyon*
Bd-C: Mock-inoculated *Bd* root sample
Bd-Si: *Si-*inoculated *Bd* root sample
*Bd*sRNA: *Brachypodium distachyon*-derived sRNA
ck-sRNA: Cross-kingdom sRNA
DCL: Dicer-like
DEG: Differentially expressed gene
DPI: Day(s) post inoculation
FC: Fold change
miRNA: MicroRNA
nt: Nucleotide
RdRP: RNA-dependent RNA polymerase
RNAi: RNA interference
*Si*: *Serendipita indica*
Si-ax: *Si* axenic culture sample
*Si*sRNA: *Serendipita indica*-derived sRNA
sRNA: Small RNA
