# Supplementary material for: A novel plant-fungal association reveals fundamental sRNA and gene expression reprogramming at the onset of symbiosis
Source: BMC Biol. 2021 Aug 24;19:171. doi: 10.1186/s12915-021-01104-2 (PMC8385953; doi:10.1186/s12915-021-01104-2)
Supplement: Supplementary file 2 — Additional file 2: Fig. S1.Serendipita indica (Si) colonization has an effect on Brachypodium distachyon (Bd) root structure. Fig. S2. Progress of Serendipita indica (Si) spore proliferation during colonization of Brachypodium distachyon Bd21-3. Fig. S3. Comparison of the resequenced Serendipita indica (Si) genome to the 2011 assembly. Fig.S4. Comparison of the resequenced Serendipita indica (Si) genome to Serendipita vermifera and Laccaria bicolor. Fig. S5. qRT-PCR confirmation of DEGs identified during mRNA sequencing. Fig. S6. Filtering pipelines applied in the analysis. Fig. S7. Size distribution of total and unique putative ck (cross-kingdom) -sRNAs. Fig. S8. Percentage distribution of the 5′ terminal nucleotide in unique putative endogenous sRNAs. Fig. S9. Percentage distribution of the 5′ terminal nucleotide in unique putative ck-sRNAs. Fig. S10. Percentage distribution of the 5′ terminal nucleotide in total putative endogenous sRNAs. Fig. S11. Percentage distribution of the 5′ terminal nucleotide in total putative ck-sRNAs. Fig. S12. Stem-loop PCR (gel electrophoresis) of some Si and Bd21-3 sRNAs expressed in the Bd-Si sample. Table S1. Quantification of identified features in the resequenced genome of Serendipita indica (Si). Table S2. Total reads from the Bd-C (mock-treated), Bd-Si (colonized root) and Si-ax (axenic culture) samples and their alignment rate (HISAT2) to the corresponding genomes. Table S3. Significant gene ontology terms of molecular function in the differentially expressed genes (DEGs) datasets. Table S4. Predicted protein effectors identified in the resequenced Serendipita indica (Si) genome. Table S5. Candidate RNAi machinery proteins predicted from the resequenced Serendipita indica (Si) genome. Table S6. Total and unique reads for filtered sRNAs from Bd-C (mock-treated), Bd-Si (colonized root) and Si-ax (axenic culture). Table S7. Sequences of putative sRNAs in Tables 4 and 5. Table S8. sRNA sequences after stem-loop PCR amplification. [file 12915_2021_1104_MOESM2_ESM.docx]

## Additional File 2: Supporting Information

## Title: A novel plant-fungal association reveals fundamental sRNA and gene expression reprogramming at the onset of symbiosis

**Authors:** Ena Šečić, Silvia Zanini, Daniel Wibberg, Lukas Jelonek, Tobias Busche, Jörn Kalinowski, Sabrine Nasfi, Jennifer Thielmann, Jafargholi Imani, Jens Steinbrenner and Karl-Heinz Kogel

**Figure S1: *Serendipita indica (Si)* colonization alters *Brachypodium distachyon* Bd21-3 root structure.** One-week-old Bd21-3 seedlings were mock treated (Bd-C) or dip-inoculated with 5 x 10^5^ chlamydospores per ml (Bd-Si) and subsequently grown for four days (a) or 25 days (b) on a vermiculite:oil dri mix.


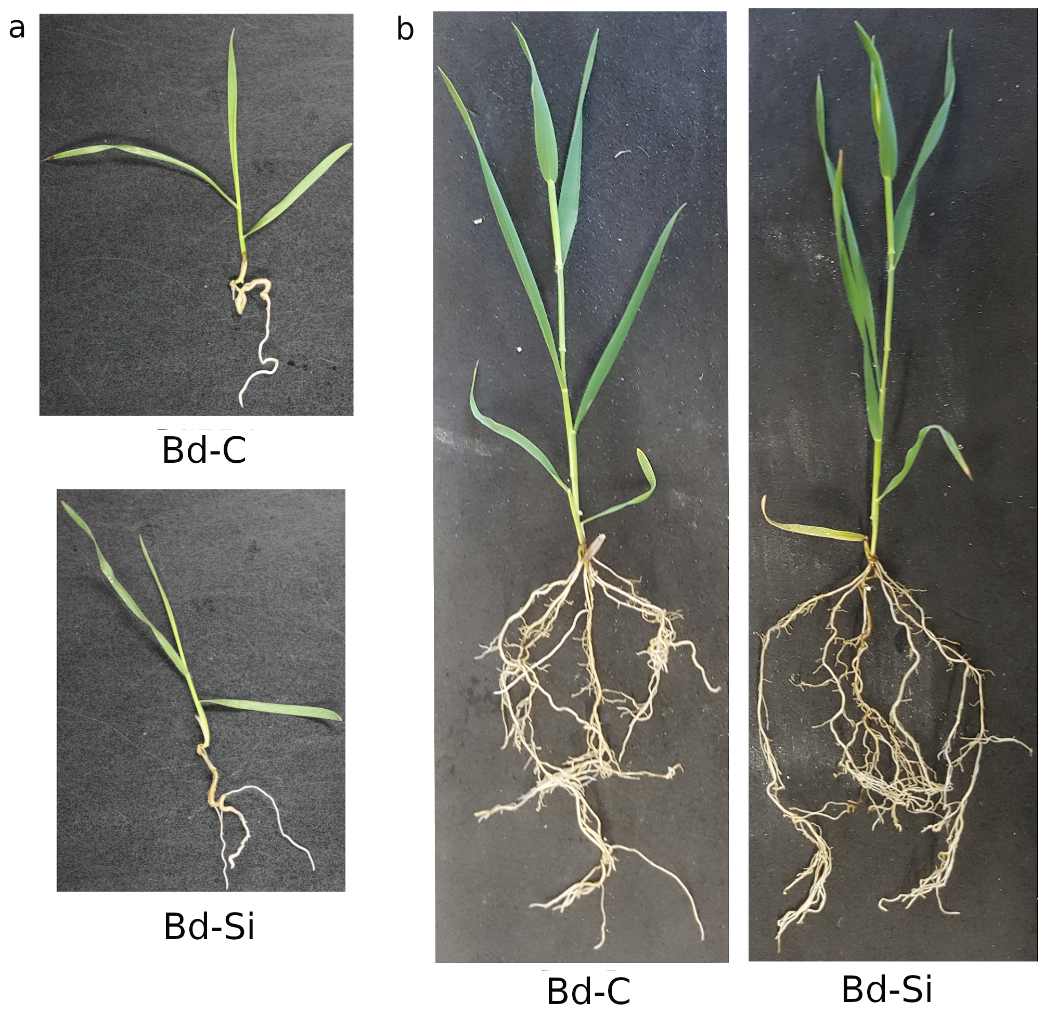


**Figure S2**: **Progress of *Serendipita indica* (*Si)* spore proliferation during colonization of *Brachypodium distachyon* Bd21-3.**  Displayed at 4DPI (days post inoculation), when new spores were first observed, and at 7DPI and 14 DPI, with an increasing number of chlamydospores. Fluorescence microscopy showing WGA-AF488 staining of *Si* cell walls (λexc494 nm, λem515). Imaging was done with a LEICA S8 confocal microscope.


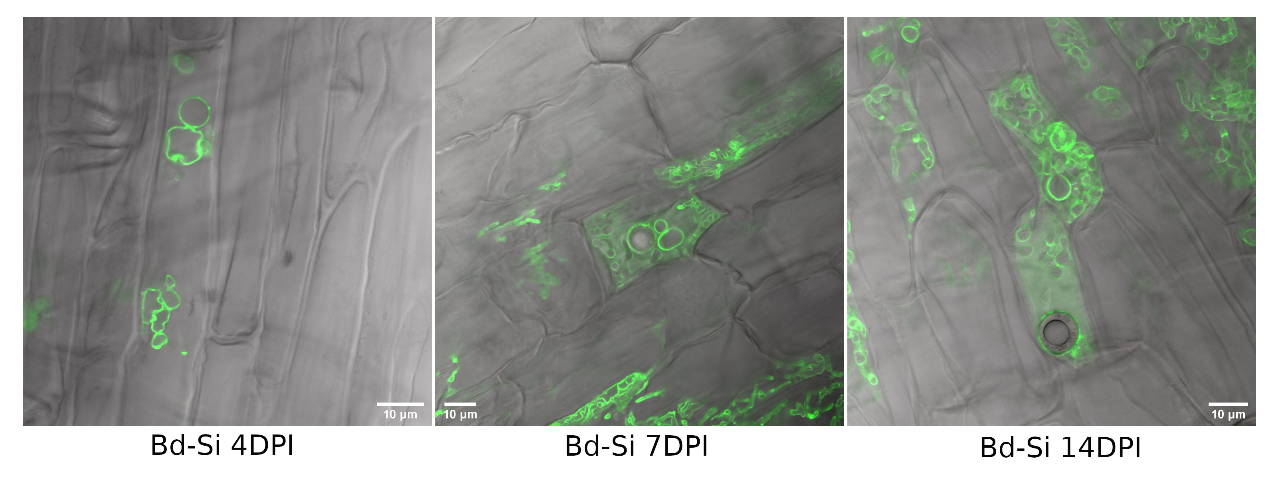


**Figure S3**: **Shared and unique genes of *Serendipita indica* between the originally sequenced and resequenced genome.** Venn diagram (as displayed by EDGAR 2.3) showing the number of genes that are shared between (core) or are unique to (single color) the resequenced *Serendipita indica* (*Si)* genome and the Zuccaro *et al.*, 2011 (22) assembly.


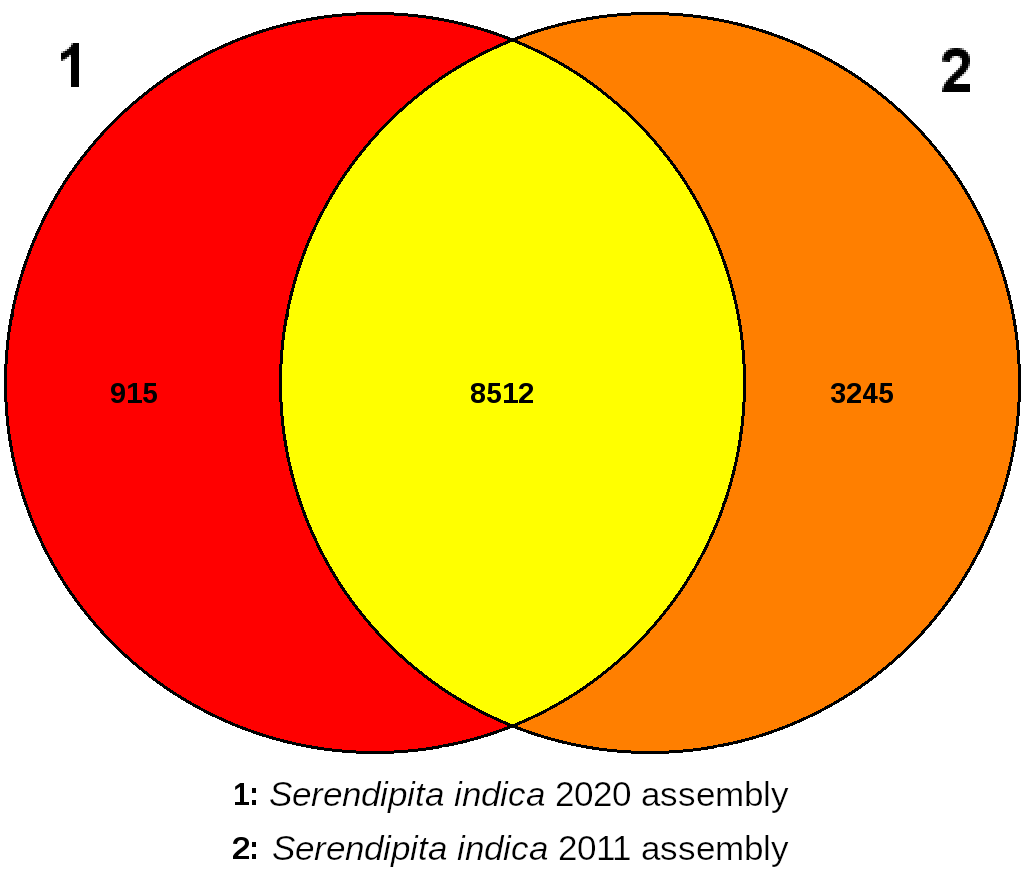


**Figure S4**: **Shared and unique genes of the *Serendipita* indica with *Serendipita vermifera and Laccaria bicolor.*** Venn diagram (as displayed by EDGAR 2.3) showing the number of genes that are shared between (core) or are unique to (single color) the resequenced *Serendipita indica* (*Si)* genome and those of *Serendipita vermifera* and *Laccaria bicolor.*


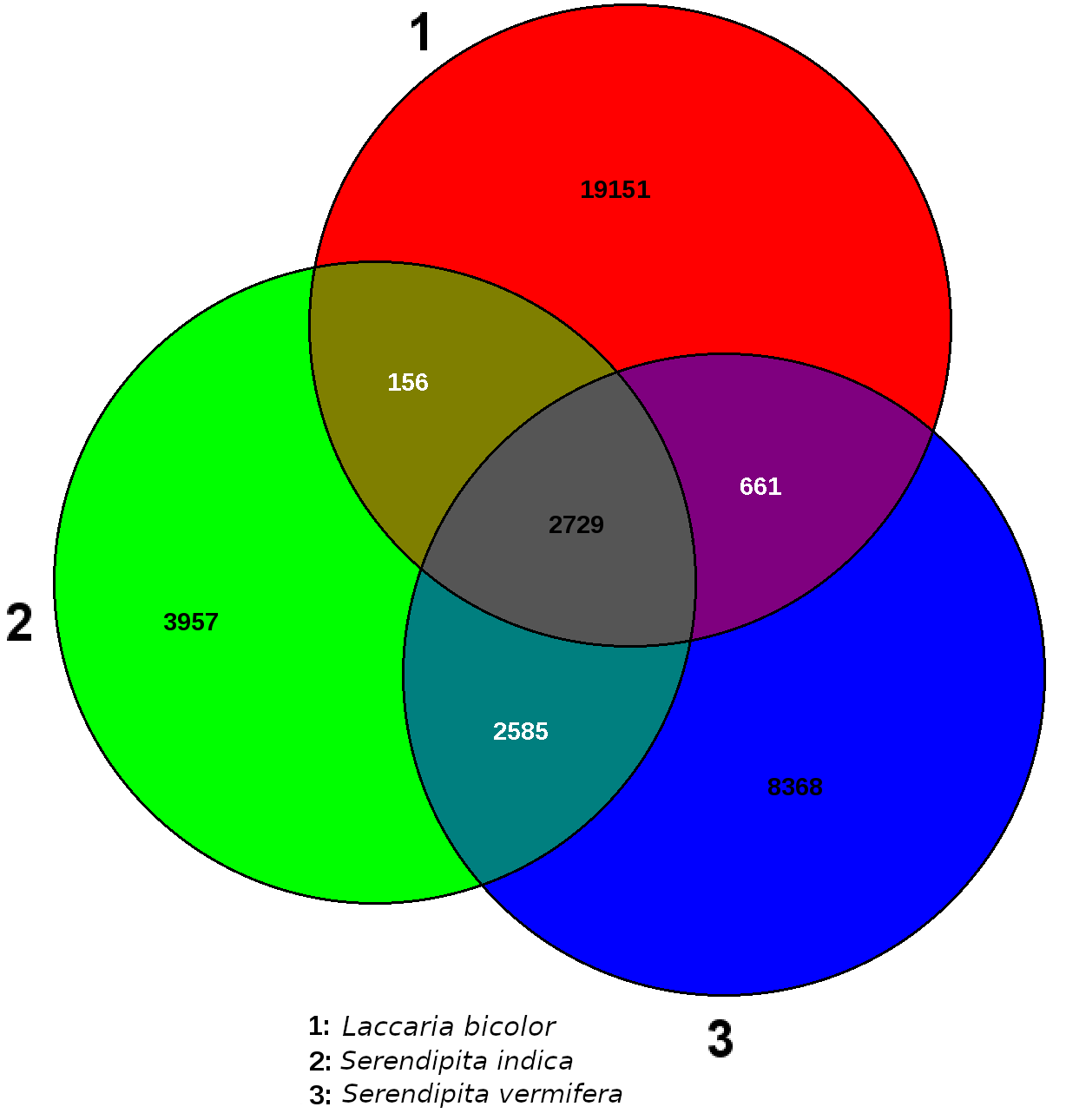


**Figure S5: Quantitative real-time PCR (qRT-PCR) of selected DEG transcripts during *Si* colonization of Bd21-3.** Expression was measured in mock-treated and *Si*-inoculated *Bd* roots (a) and axenic *Si* control and *Si*-inoculated *Bd* roots (b). One-week-old seedlings were inoculated with 5 x 10^5^ chlamydospores per ml and subsequently grown for four days on a vermiculite:oil dri mixture (semi-sterile conditions). Sample size (n) = 3 biological samples, each containing 3-4 pooled roots. Two independent biological replicates (inoculations) were performed for each gene, standard error (SE) bars indicated. Individual data values of fold change are available in Additional File 1. The significance threshold after t-test or Mann-Whitney-Wilcoxon test was set at .05 (*=<.05;**=<.001). Bd transcripts tested (in Table 2): glycosyl-hydrolase (BdiBd21-3.3G0639500.1), HSP 90-1 (BdiBd21-3.5G0024800.1), PRR7 (BdiBd21-3.1G0887100.1), RCI2 (BdiBd21-3.1G0416000.1), WAK (BdiBd21-3.2G0600500.1). Primers used in Table S9.


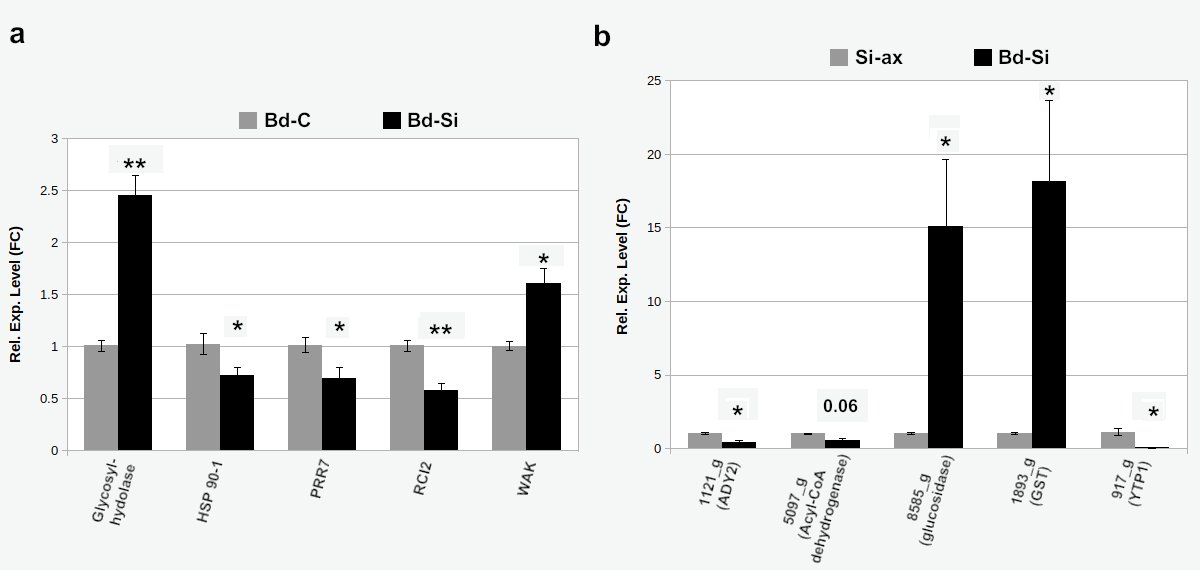


**Figure S6**: **Filtering pipelines utilized in sRNA analysis.** These pipelines were used to identify putative cross-kingdom (ck-sRNAs) and putative endogenous sRNAs in *Serendipita indica* (*Si)* and *Brachypodium distachyon* Bd21-3.


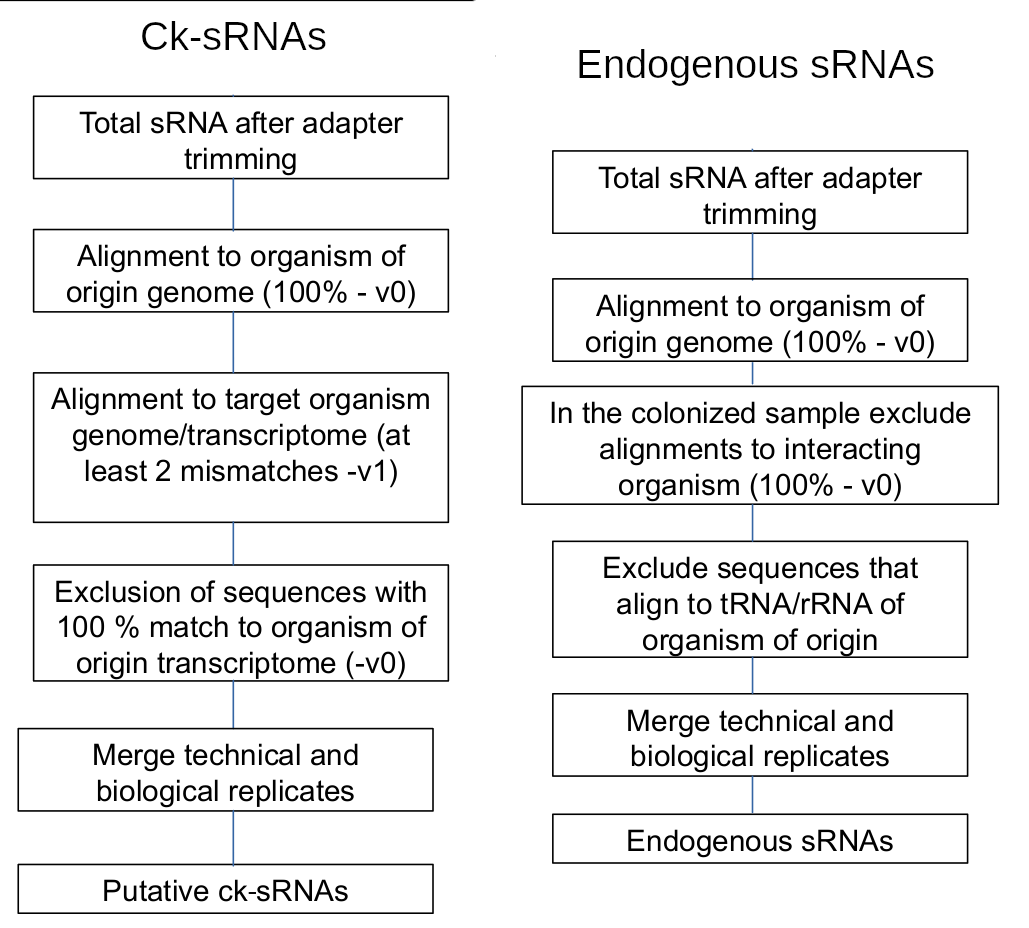


**Figure S7**: **Size distribution of total and unique putative ck-sRNAs in the interaction and reads from controls.** a) Bd-C (mock-treated), b) Bd-Si (colonized root), c) Si-ax (axenic *Si* culture), and d) Bd-Si. All datasets represent three biological replicates and corresponding two technical replicates, merged together. sRNA length is displayed on the X-axis (nt) and number of total/unique sRNA counts on the Y-axis (x 10^3^).


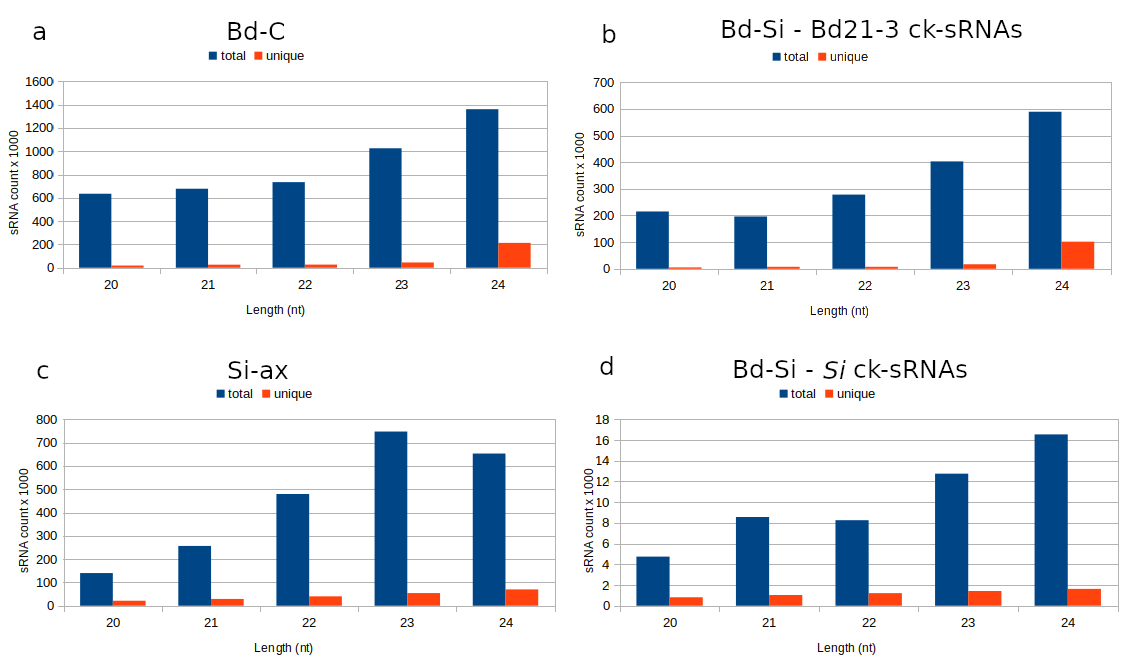


**Figure S8: 5’ terminal nucleotide distribution of unique putative endogenous sRNAs.** Percentage distribution of the 5’ terminal nucleotide in unique putative endogenous sRNAs (18 – 30 nt) from Bd-C (mock-treated), Bd-Si (colonized root) and Si-ax (axenic culture). *Brachypodium distachyon* Bd21-3- and *Serendipita indica*-associated endogenous sRNAs were identified from the colonized root sample by aligning filtered sRNAs with the plant or fungal genome; they are presented as Bd-Si Bd endg or Bd-Si Si endg, respectively.


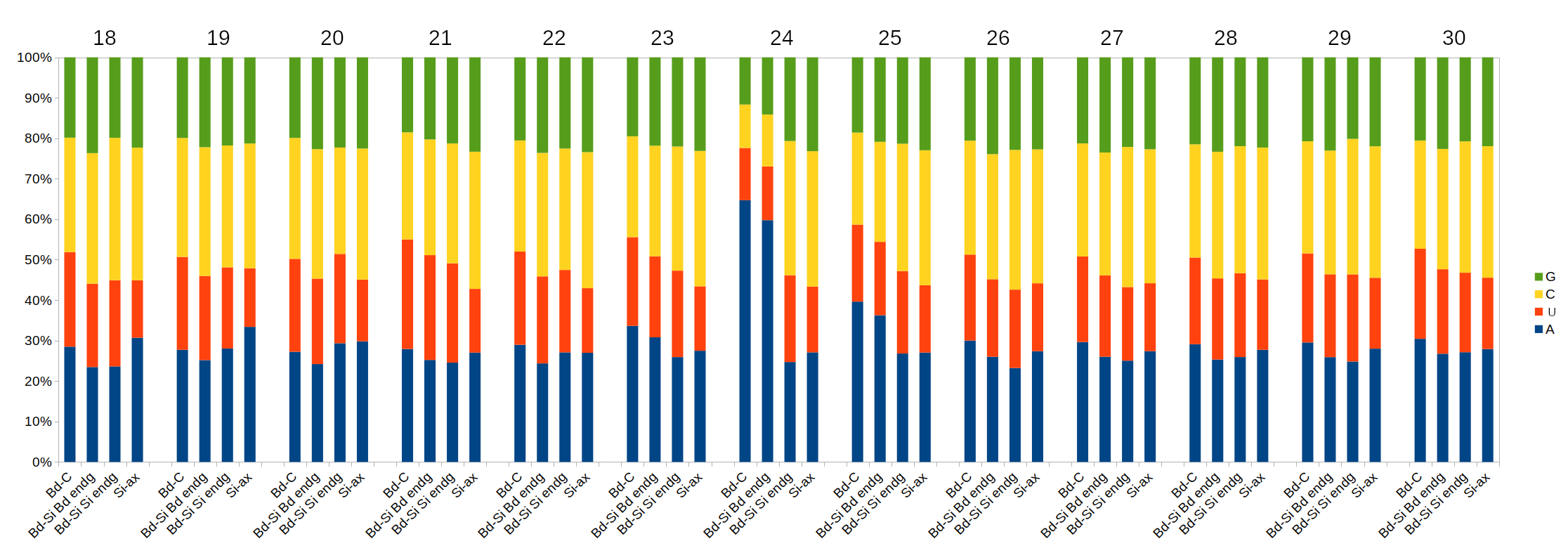


**Figure S9: 5’ terminal nucleotide distribution of unique putative ck-sRNAs.** Percentage distribution of the 5’ terminal nucleotide in unique reads (20-24 nt long) from Bd-C (mock-treated) and Si-ax (axenic culture) samples, putative ck-sRNAs from Bd-Si (colonized root) sample. *Brachypodium distachyon* Bd21-3- and *Serendipita indica*-associated ck-sRNAs were identified from the colonized root sample by aligning filtered sRNAs with the plant or fungal genome; they are presented as Bd-Si Bd ck or Bd-Si Si ck, respectively.


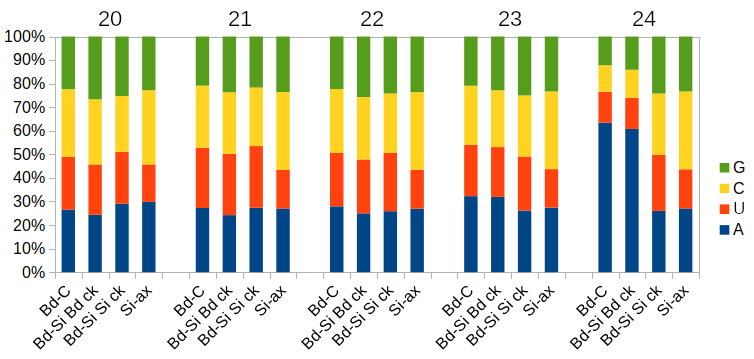


**Figure S10**: **5’ terminal nucleotide distribution of total putative endogenous sRNAs.** Percentage distribution of the 5’ terminal nucleotide in total putative endogenous sRNAs (18 – 30 nt long) from Bd-C (mock-treated), Bd-Si (colonized root) and Si-ax (axenic culture). *Brachypodium distachyon* Bd21-3- and *Serendipita indica*-associated endogenous sRNAs were identified from the colonized root sample by aligning filtered sRNAs with the plant or fungal genome; they are presented as Bd-Si Bd endg or Bd-Si Si endg, respectively.


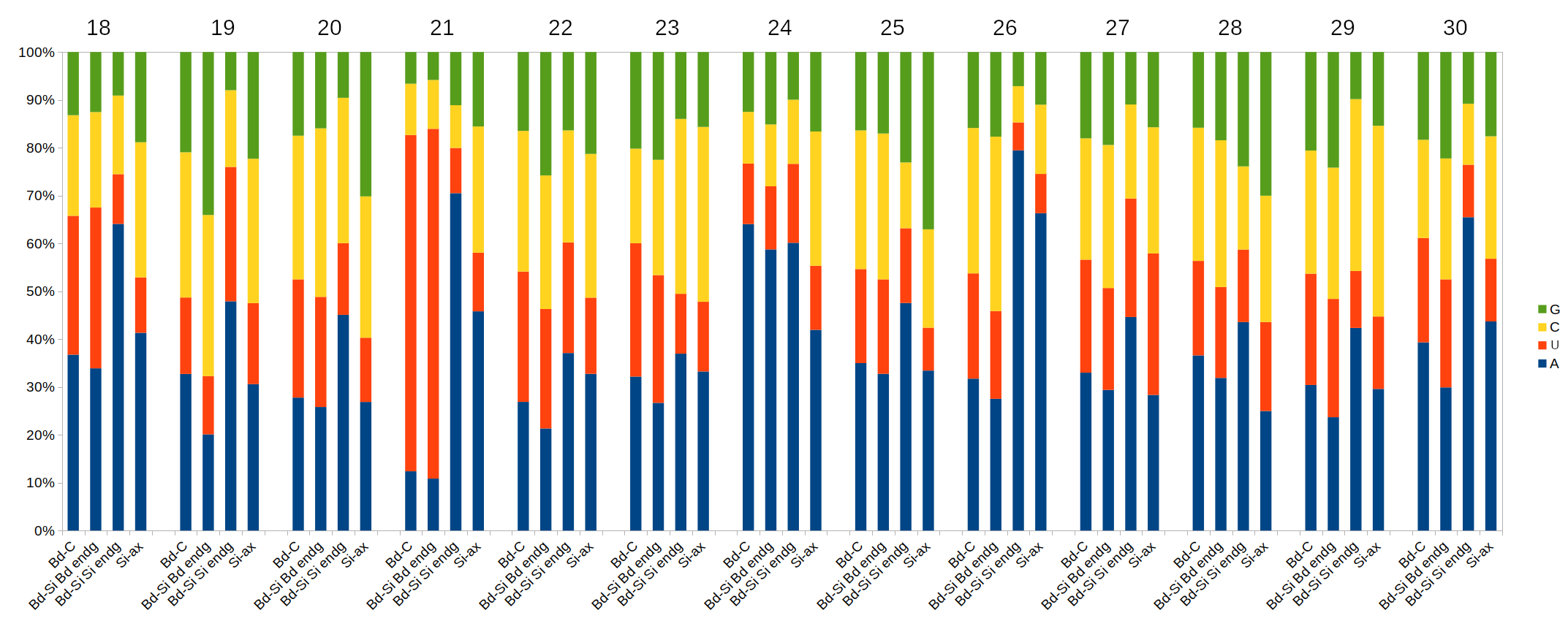


**Figure S11**: **5’ terminal nucleotide distribution of total putative ck-sRNAs.** Percentage distribution of the 5’ terminal nucleotide in total reads (20-24 nt long) from Bd-C (mock-treated) and Si-ax (axenic culture) samples, putative ck-sRNAs from Bd-Si (colonized root) sample. *Brachypodium distachyon* Bd21-3- and *Serendipita indica*-associated ck-sRNAs were identified from the colonized root sample by aligning filtered sRNAs with the plant or fungal genome; they are presented as Bd-Si Bd ck or Bd-Si Si ck, respectively.


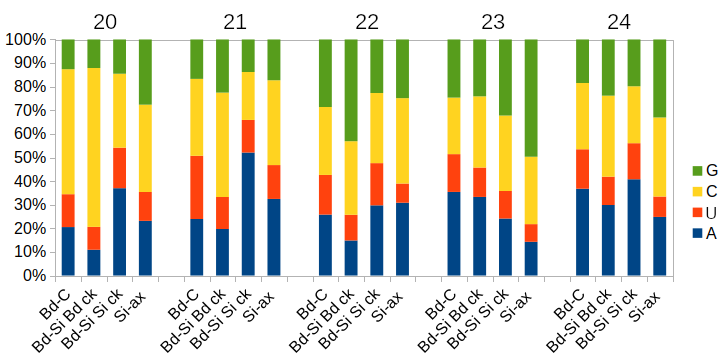


**Figure S12: Stem-loop PCR to show expression of selected sRNAs during the interaction.** Gel electrophoresis after stem-loop PCR of *Si* (a) and Bd21-3 (b) sRNAs identified in the Bd-Si sample during sequencing. Length of amplicon (sRNA+hairpin) is 65bp. Small RNAs marked with (*) were further confirmed by sequencing (Table S8), and the primers used are displayed in Table S10. Original gel pictures are available as Additional File 3 and Additional File 4.


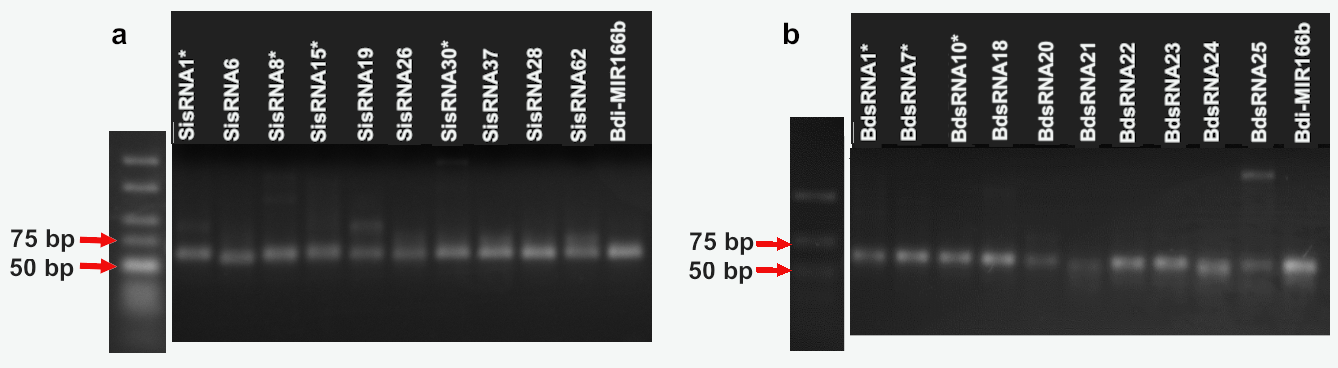


**Table S1**: Quantification of identified features in the resequenced genome of *Serendipita indica (Si).*

| **Genome feature** | **Value** |
| --- | --- |
| Gene density | 380.68 genes/Mbp |
| Contigs | 57 |
| Total Genome Size | 24.8Mbp (24724951 bp) |
| Gene/mRNA number | 9441 |
| Exon number | 59045 |
| Intron number | 49604 |
| Intergenic region | 9498 |
| Mean (median) gene length | 1955.36bp (1554.00bp) |
| Mean (median) exon length | 259.97bp (149.00bp) |
| Mean (median) intron length | 62.71bp (48.00bp) |
| Mean (median) intergenic region | 659.54bp (363.00bp) |
| Exons/Gene | 6.25 |
| Cumulative length of genes (% of genome) | 18.5M bp (18460589 bp) (0.75) |
| Cumulative length of exons (% of genome) | 15.4M bp (15350115 bp) (0.62) |
| Cumulative length of introns (% of genome) | 3.2M bp (3110474 bp) (0.13) |
| Cumulative length of intergenic regions (% of genome) | 6.3M bp (6264362 bp) (0.25) |

**Table S2**: Total reads from the Bd-C (mock-treated), Bd-Si (colonized root) and Si-ax (axenic culture) samples.

| **Sample description** | **Total reads (PE)** | **Overall alignement rate to genome** |
| --- | --- | --- |
| *Si* axenic culture (Si-ax) | 53 million | 96.54 % reads align to *Si* genome |
| Bd21-3 mock treated control (Bd-C) | 53.2 million | 98 % reads align to Bd21-3 genome |
| Bd21-3 + *Si* 4 DPI (Bd-Si) | 55 million | 96.55 % reads align to Bd21-3 genome |
|  |  | 2.13 % reads align to *Si* genome |

**Table S3**: Significant GO terms of molecular function in the differentially expressed gene (DEG) datasets.*

| Term ID | P-value (FDR) | Term |
| --- | --- | --- |
| **Si DEGs** | | |
| GO:0016491 | 0.00007 | oxidoreductase activity |
| GO:0004553 | 0.00007 | hydrolase activity hydrolyzing O-glycosyl compounds |
| GO:0016798 | 0.00007 | hydrolase activity acting on glycosyl bonds |
| GO:0030246 | 0.00007 | carbohydrate binding |
| GO:0030247 | 0.00007 | polysaccharide binding |
| GO:0001871 | 0.00007 | pattern binding |
| GO:0030248 | 0.00007 | cellulose binding |
| GO:0016876 | 0.00007 | ligase activity forming aminoacyl-tRNA and related compounds |
| GO:0004812 | 0.00007 | aminoacyl-tRNA ligase activity |
| GO:0016875 | 0.00007 | ligase activity forming carbon-oxygen bonds |
| GO:0003824 | 0.00017 | catalytic activity |
| GO:0016874 | 0.00028 | ligase activity |
| GO:0003995 | 0.0055 | acyl-CoA dehydrogenase activity |
| GO:0048037 | 0.01 | cofactor binding |
| GO:0016627 | 0.01 | oxidoreductase activity acting on the CH-CH group of donors |
| GO:0016684 | 0.012 | oxidoreductase activity acting on peroxide as acceptor |
| GO:0004601 | 0.012 | peroxidase activity |
| GO:0016829 | 0.019 | lyase activity |
| GO:0016620 | 0.028 | oxidoreductase activity acting on the aldehyde or oxo group of donors NAD or NADP as acceptor |
| GO:0070003 | 0.028 | threonine-type peptidase activity |
| GO:0004298 | 0.028 | threonine-type endopeptidase activity |
| GO:0002161 | 0.033 | aminoacyl-tRNA editing activity |
| GO:0016903 | 0.04 | oxidoreductase activity acting on the aldehyde or oxo group of donors NAD or NADP as acceptor |
| GO:0000287 | 0.044 | magnesium ion binding |
| GO:0050662 | 0.045 | coenzyme binding |
| GO:0016840 | 0.045 | carbon-nitrogen lyase activity |
| GO:0004725 | 0.047 | protein tyrosine phosphatase activity |
| **Bd21-3 DEGs** | | |
| GO:0046906 | 0.00000039 | tetrapyrrole binding |
| GO:0020037 | 0.00000039 | heme binding |
| GO:0003824 | 0.00000072 | catalytic activity |
| GO:0016829 | 0.000016 | lyase activity |
| GO:0005506 | 0.000018 | iron ion binding |
| GO:0016491 | 0.000018 | oxidoreductase activity |
| GO:0016705 | 0.000035 | oxidoreductase activity acting on paired donors with incorporation or reduction of molecular oxygen |
| GO:0048037 | 0.0036 | cofactor binding |
| GO:0016830 | 0.0039 | carbon-carbon lyase activity |
| GO:0016831 | 0.0066 | carboxy-lyase activity |
| GO:0043168 | 0.014 | anion binding |
| GO:0016684 | 0.014 | oxidoreductase activity acting on peroxide as acceptor |
| GO:0004601 | 0.014 | peroxidase activity |
| GO:0030170 | 0.016 | pyridoxal phosphate binding |
| GO:0016209 | 0.017 | antioxidant activity |
| GO:0016887 | 0.017 | ATPase activity |
| GO:0016758 | 0.027 | transferase activity transferring hexosyl groups |
| GO:0043167 | 0.031 | ion binding |
| GO:0016757 | 0.034 | transferase activity transferring glycosyl groups |

* Analysis was done on *Serendipita indica* and *Brachypodium distachyon* Bd21-3 with AgriGO v2.0. Hochberg (FDR) adjustment for multiple testing was used and is reported in the table.

**Table S4**: A representative set of potential protein effectors identified in the resequenced *Serendipita indica* *(Si)* genome.*

| **Putative effector gene** | **log2FC** | **Description** | **DE putative effector** |
| --- | --- | --- | --- |
| 5323_g(PIIN_05891) | 13.26 | Hypothetical protein |  |
| 2999_g(PIIN_05674) | 10.99 | Hypothetical protein |  |
| 7143_g(PIIN_07595) | 10.53 | Hypothetical protein, DELD | *Hv* colonization |
| 4184_g(PIIN_06901) | 9.16 | Hypothetical protein |  |
| 3615_g(PIIN_11270) | 7.36 | Related to esterase D |  |
| 5279_g(PIIN_10706) | 7.36 | Hypothetical protein, DELD | *Hv* and *At* colonization |
| 3514_g(PIIN_09664) | 7.01 | Related to glucose oxidase |  |
| 2829_g(PIIN_07952) | 6.66 | Hypothetical protein |  |
| 2677_g(PIIN_08766) | 6.5 | Related to hydrophobin |  |
| 6427_g(PIIN_07750) | 6.42 | Related to triacylglycerol lipase precursor |  |
| 7044_g(PIIN_07884) | 6.38 | Hypothetical protein |  |
| 4610_g(PIIN_01386) | 6.36 | Hypothetical protein |  |
| 4303_g(PIIN_03861) | 6.21 | Hypothetical protein |  |
| 7637_g(PIIN_08723) | 6.2 | Hypothetical protein, lectin-like LysM | *Hv* colonization |
| 3661_g(PIIN_09859) | 6.18 | Hypothetical protein, DELD | *Hv* and *At* colonization |
| 6698_g(PIIN_06410) | 6.18 | Hypothetical protein |  |
| 6458_g(PIIN_05098) | 5.71 | Hypothetical protein, DELD | *Hv* and *At* colonization |
| 7883_g(PIIN_05242) | 5.6 | Related to peptidyl-Lys metalloendopeptidase |  |
| 9216_g(PIIN_03736) | 5.5 | Probable acetylxylan esterase |  |
| 6575_g(PIIN_01273) | 4.9 | Hypothetical protein |  |
| 7037_g(PIIN_08942) | 4.78 | Related to metalloprotease |  |
| 4183_g(PIIN_06900) | 4.75 | Hypothetical protein |  |
| 6634_g(PIIN_03075) | 4.58 | Hypothetical protein |  |
| 767_g(PIIN_08824) | 4.23 | Related to WD40-repeat protein (notchless protein) |  |
| 7142_g(N/A) | 3.91 | Hypothetical protein, DELD |  |
| 5200_g(PIIN_07639) | 3.9 | Hypothetical protein |  |
| 5275_t(PIIN_06837) | 4.02 | Hypothetical protein, DELD | *Hv* and *At* colonization |
| 3238_g(PIIN_09643) | 1.9 | PiDld2 | *Hv* and *At* colonization |
| 6196_g(PIIN_04163) | 1.44 | Related to HSP82-Heat shock protein |  |
| 8967_g(PIIN_03461) | 6.09 | Hypothetical protein |  |
| 2675_g(PIIN_08764) | 2.66 | Probable ADE12-adenylosuccinate synthetase |  |
| 8386_g(PIIN_00425) | 0.91 | Probable extracellular elastinolytic metalloproteinase precursor |  |

*Those with the highest level of upregulation during Bd21-3 colonization are displayed. Prior detection of differentially expressed (DE) putative effector proteins in *Si*-colonized barley (*Hordeum vulgare - Hv*) or Arabidopsis (*At*) is indicated (22, 27).

**Table S5:** Candidate RNAi machinery proteins predicted from the resequenced *Serendipita indica (Si)* genome.*

| Gene | Protein | Description / Domains | *N. crassa* homolog | Score (E-value) / Identity (BlastP) | baseMean Deseq2 |
| --- | --- | --- | --- | --- | --- |
| 1587_g (PIIN_03414) | G4U2H0 | Dicer-like protein, Helicase, dicer ds-RNA binding, RNAse III | NCU08270 | 330 (3E-52) / 30.71 % | 54.36 |
| 7403_g (PIIN_02669) | G4TBW9 |  |  | 273 (9E-75) / 24.15 % | 51.81 |
| 5425_g (PIIN_06945) | G4TNU7 | Related to qde-1 RNA-dependent RNA polymerase (RdRP) | NCU07534 | 111(9E-25)/25.97 % | 102.65 |
| 2067_g (PIIN_07582) | G4TQP0 |  |  | 92.4 (5E-19) / 30.32 % | 49.21 |
| 3379_g (PIIN_03690) | G4TEK0 | QDE2 – like protein, PIWI domain | NCU04730 | 354 (2E-106) / 36.26 % | 16.05 |
| 3261_g (PIIN_05928) | G4TL04 |  |  | 224 (1E-61) / 31.05 % | 2.11 |
| 5826_g (PIIN_00261) | G4T5G9 | Related to argonaute-like protein-*Laccaria bicolor* |  | 233 (5E-64) / 28.59 % | 124.7 |
| 1551_g (PIIN_06323) | G4TM47 | Related to QDE3 protein, helicase domains | NCU08598 | 339 (3E-101) / 44.5 % | 47.47 |
| 5685_g (PIIN_06751) | G4TND7 | Probable DNA-directed RNA polymerase I | NCU01638 | 1208 (0.0) / 41.22 % | 60.38 |

* Homology with quelling-typical *Neurospora crassa* proteins is indicated. The baseMean (Deseq2; 107) is presented as an average of normalized count values across all samples.

**Table S6:** Total and unique reads for filtered sRNAs from Bd-C, Bd-Si and Si-ax.*

| sRNA dataset | Description | Number of sRNA |
| --- | --- | --- |
| Si-ax | Total reads after adapter trimming | 48 mil |
|  | Total sRNAs, 100 % alignment to Si genome | 33.9 mil |
|  | Unique sRNAs, 100 % alignment to Si genome | 1.9 mil |
|  | Total endogenous sRNAs | 11 mil |
|  | Unique endogenous sRNAs | 1.8 mil |
| Bd-Si | Total reads after adapter trimming | 32.7 mil |
| *Si* → Bd21-3 | Total putative ck sRNAs | 786732 |
|  | Unique putative ck sRNAs | 35895 |
| Bd21-3 → *Si* | Total putative ck sRNAs | 17 mil |
|  | Unique putative ck sRNAs | 286198 |
| *Si* aligned sRNAs | Total endogenous sRNAs | 261478 |
|  | Unique endogenous sRNAs | 36163 |
| Bd21-3 aligned sRNAs | Total endogenous sRNAs | 1.6 mil |
|  | Unique endogenous sRNAs | 483352 |
| Bd-C | Total reads after adapter trimming | 55 mil |
|  | Total sRNAs, 100 % alignment to Bd21-3 genome | 34.6 mil |
|  | Unique sRNAs, 100 % alignment to Bd21-3 genome | 1 mil |
|  | Total endogenous sRNAs | 3 mil |
|  | Unique endogenous sRNAs | 906901 |

**Table S7:** Sequences of putative *Serendipita indica* and *Brachypodium distachyon* Bd21-3 ck-sRNA displayed in Table 4 and Table 5.

| **sRNA name** | **sRNA sequence** |
| --- | --- |
| *Si*sRNA 1 | AACUUCUCGAAUCGCAUGGCC |
|  |  |
| *Si*sRNA 2 | AAUGCUGGGUGGAACGGCCCC |
| *Si*sRNA 3 | AGCACCGCAUCCCGUCCGAUC |
| *Si*sRNA 4 | AUGAAAGUGAAAGGUCGGGAA |
| *Si*sRNA 5 | AUGGUUAGGAUUUGUCGCUCU |
| *Si*sRNA 6 | CAAACCCGGACGCGUAAUGAA |
| *Si*sRNA 7 | CCAUAGGACUCUGAAAGCACC |
| *Si*sRNA 8 | CGAGUAGGCAGGCGUGGGGGU |
| *Si*sRNA 9 | CUCGAAUCGCAUGGCCUUGUG |
| *Si*sRNA 10 | CUUAUUUUUGACGCCGUCGCC |
| *Si*sRNA 11 | GACGCAUCCGCGGUAGAGCGU |
| *Si*sRNA 12 | GAGGGAAAAAGGCUUGGGGAA |
| *Si*sRNA 13 | GCACCGCAUCCCGUCCGAUCU |
| *Si*sRNA 14 | GCCAUAGGACUCUGAAAGCAC |
| *Si*sRNA 15 | GGGUCCGGUGCGUCCUCGACA |
|  |  |
| *Si*sRNA 16 | GGUUCGAUUCCCCGAUUCGGA |
| *Si*sRNA 17 | GUAAGCGUUGGAUUGUUCACC |
| *Si*sRNA 18 | UCCGGCAACGGAACCCCGUUG |
| *Si*sRNA 19 | UUGGGGAUCCGGCAACGGAAC |
| *Si*sRNA 20 | UUGUCGUGCUGGGGAUAGAGC |
| *Bd*sRNA_1 | ACGGUGGUCAUGGAAGUCGAA |
| *Bd*sRNA_2 | ACUGUUGUCGGCCGUGCUGGC |
| *Bd*sRNA_3 | AGCCCCGUCCGGCCCGGACCC |
| *Bd*sRNA_4 | AGUUAAGCGUGCUUGGGCGAG |
| *Bd*sRNA_5 | CCGGGCGGAGCGGCCGUCGGU |
| *Bd*sRNA_6 | CCUGGACGGGGUCUAUGGCCG |
| *Bd*sRNA_7 | CGGGGGACGGACCGGGAGUCG |
| *Bd*sRNA_8 | CGUGCUUGGGCGAGAGUAGUA |
|  |  |
| *Bd*sRNA_9 | CUGACAUGCGUGCGAGUCGAC |
| *Bd*sRNA_10 | CUGCACUGCCUCUUCCCUGGC |
|  |  |
|  |  |
| *Bd*sRNA_11 | CUUAUUCCGUGGGUCGGAAGC |
| *Bd*sRNA_12 | CUUUGAACGCAAGUUGCGCCC |
| *Bd*sRNA_13 | GAGUCGACGGGUUCUGAAACC |
| *Bd*sRNA_14 | GCCACCCGGUCGAGGGCACGC |
| *Bd*sRNA_15 | GCGAGUCGACGGGUUCUGAAA |
| *Bd*sRNA_16 | GCUCGUAGUUGGACUUUGGGC |
| *Bd*sRNA_17 | GGCCGUGCCGUCCGAAUUGUA |
| *Bd*sRNA_18 | GGCGAGCGAACCGGGAGCAGC |
| *Bd*sRNA_19 | GGCUUUUGCUGGUCCGCCGCU |
|  |  |
| *Bd*sRNA_20 | UCGAGUCUUUGAACGCAAGUU |

* Putative endogenous and ck-sRNAs were identified via the filtering pipelines shown in Figure S6.

**Table S8**: Sequencing results from stem-loop PCR amplification of the selected *Si* and *Bd* sRNAs.*

* Performed after cloning into pGEMT-Easy vector (Figure S12). Small RNA sequence in the sequencing results is underlined and the stem-loop hairpin is in bold.

**Table S9**: List of primers used for qPCR validation of DEGs from mRNA sequencing.

| Primer name | Sequence |
| --- | --- |
| Ubi4-3Fw | GCTGTTGGAACTGCTGCTATACCT |
| Ubi4-3Rv | TTGCACCAAACCAACACACACCAG |
| Bd213_PRR7_F | CAGTTGATGGTGGGTGCCT |
| Bd213_PRR7_R | TCGTCATTACCGCTCCTCCA |
| Bd213_PR5_F | CTACGTGTGCGAGCAGTCTT |
| Bd213_PR5_R | CTTTGAGTGTGCCCTTGATGC |
| Bd213_HSP90_F | TGAGGTTCAGAGTTGGTCGC |
| Bd213_HSP90_R | CCCGAGCCTTTGGATCACTT |
| Bd213_Chit_rel_F | CGTCGCCTATTACGAGCAGT |
| Bd213_Chit_rel_R | ATGCCACAAATCAAACGCCC |
| Bd213_RCI2_F | CCTGTGGTCGCCAATGACAC |
| Bd213_RCI2_R | CGCAGGAGAGCCTATTCGTT |
| Piri_ITS_F | CAACACATGTGCACGTCGAT |
| Piri_ITS_R | CCAATGTGCATTCAGAACGA |
| 1121_t_ADY2_F | CCACCACGACTACCCAACAA |
| 1121_t_ADY2_R | AGGACCAGGATTGCCAAGTG |
| 5097_t_acCOdh_F | CGGTGTCTTTGCCGACTACT |
| 5097_t_acCOdh_R | ACTGGGACCTTGACGTTGTC |
| 917_t_YTP1_F | GCAAACATCCAGGAGACCCA |
| 917_t_YTP1_R | CCAGCCCAAAACATGACAGC |
| 8585_t_glucosidase_F | CTCACCGTCCTCGACAACAA |
| 8585_t_glucosidase_R | CCCCGCTTCAATCTTCCACT |
| 1893_t_GST_F | ATGAGCTGACCTATGCCGAC |
| 1893_t_GST_R | TTCCTCCCTTGGCTTTCTGC |

**Table S10**: Hairpin primers and specific primers used in stem-loop PCR confirmation of sequencing-detected sRNAs.

| hairpin Primer name | hairpin Primer sequence |
| --- | --- |
| qPSisRNA1_cDNAhp | GTCGTATCCAGTGCAGGGTCCGAGGTATTCGCACTGGATACGACggccat |
| qPSisRNA6_cDNAhp | GTCGTATCCAGTGCAGGGTCCGAGGTATTCGCACTGGATACGACttcatt |
| qPSisRNA8_cDNAhp | GTCGTATCCAGTGCAGGGTCCGAGGTATTCGCACTGGATACGACaccccc |
| qPSisRNA15_cDNAhp | GTCGTATCCAGTGCAGGGTCCGAGGTATTCGCACTGGATACGACtgtcga |
| qPSisRNA19_cDNAhp | GTCGTATCCAGTGCAGGGTCCGAGGTATTCGCACTGGATACGACgttccg |
| qPSisRNA26_cDNAhp | GTCGTATCCAGTGCAGGGTCCGAGGTATTCGCACTGGATACGACgatccg |
| qPSisRNA30_cDNAhp | GTCGTATCCAGTGCAGGGTCCGAGGTATTCGCACTGGATACGACgccgac |
| qPSisRNA37_cDNAhp | GTCGTATCCAGTGCAGGGTCCGAGGTATTCGCACTGGATACGACacaaat |
| qPSisRNA28_cDNAhp | GTCGTATCCAGTGCAGGGTCCGAGGTATTCGCACTGGATACGACagatcc |
| qPSisRNA62_cDNAhp | GTCGTATCCAGTGCAGGGTCCGAGGTATTCGCACTGGATACGACcagagt |
| qPBdsRNA1_cDNAhp | GTCGTATCCAGTGCAGGGTCCGAGGTATTCGCACTGGATACGACttcgac |
| qPBdsRNA7_cDNAhp | GTCGTATCCAGTGCAGGGTCCGAGGTATTCGCACTGGATACGACcgactc |
| qPBdsRNA10_cDNAhp | GTCGTATCCAGTGCAGGGTCCGAGGTATTCGCACTGGATACGACgccagg |
| qPBdsRNA18_cDNAhp | GTCGTATCCAGTGCAGGGTCCGAGGTATTCGCACTGGATACGACgctgct |
| qPBdsRNA_20_cDNAhp | GTCGTATCCAGTGCAGGGTCCGAGGTATTCGCACTGGATACGACaacttg |
| qPBdsRNA_21_cDNAhp | GTCGTATCCAGTGCAGGGTCCGAGGTATTCGCACTGGATACGACgccggc |
| qPBdsRNA_22_cDNAhp | GTCGTATCCAGTGCAGGGTCCGAGGTATTCGCACTGGATACGACaggaat |
| qPBdsRNA_23_cDNAhp | GTCGTATCCAGTGCAGGGTCCGAGGTATTCGCACTGGATACGACgccggc |
| qPBdsRNA_24_cDNAhp | GTCGTATCCAGTGCAGGGTCCGAGGTATTCGCACTGGATACGACgccagc |
| qPBdsRNA_25_cDNAhp | GTCGTATCCAGTGCAGGGTCCGAGGTATTCGCACTGGATACGACgaggcc |
| qPBdi-MIR166b_cDNAhp | GTCGTATCCAGTGCAGGGTCCGAGGTATTCGCACTGGATACGACggggaa |
| Specific forward primer name | specific forward primer sequence |
| qPSisRNA1_F | TCGCTaacttctcgaatcgc |
| qPSisRNA6_F | TCGCTcaaacccggacgcgt |
| qPSisRNA8_F | TCGCTcgagtaggcaggcgt |
| qPSisRNA15_F | TCGCTgggtccggtgcgtcc |
| qPSisRNA19_F | TCGCTttggggatccggcaa |
| qPSisRNA26_F | TCGCTtacaactttcaacaa |
| qPSisRNA30_F | TCGCTtacccatacctcgcc |
| qPSisRNA37_F | TCGCTgctcacgttctatag |
| qPSisRNA28_F | TCGCTacaactttcaacaac |
| qPSisRNA62_F | TCGCTatccacggccatagg |
| qPBdsRNA1_F | TCGCTacggtggtcatggaa |
| qPBdsRNA7_F | TCGCTcgggggacggaccgg |
| qPBdsRNA10_F | TCGCTctgcactgcctcttc |
| qPBdsRNA18_F | TCGCTggcgagcgaaccggg |
| qPBdsRNA20_F | TCGCTtcgagtctttgaacg |
| qPBdsRNA_21_F | TCGCTagcgggtcgccgcgt |
| qPBdsRNA_22_F | TCGCTaagtcctcgtgttgc |
| qPBdsRNA_23_F | TCGCTtgcttataggactcc |
| qPBdsRNA_24_F | TCGCTactgttgtcggccgt |
| qPBdsRNA_25_F | TCGCTggagacgccggcggg |
| qPBdi-MIR166b_F | TCGCTtcggaccaggcttca |
| primer name | primer sequence |
| M13_F | GTTTTCCCAGTCACGAC |
| M13_R | AACAGCTATGACCATG |
| univ_stemloop_PCR_rev | GTATCCAGTGCAGGGTCCGAGGT |
