# Supplementary figures and images for: A novel plant-fungal association reveals fundamental sRNA and gene expression reprogramming at the onset of symbiosis
Source: BMC Biol. 2021 Aug 24;19:171. doi: 10.1186/s12915-021-01104-2 (PMC8385953; doi:10.1186/s12915-021-01104-2)

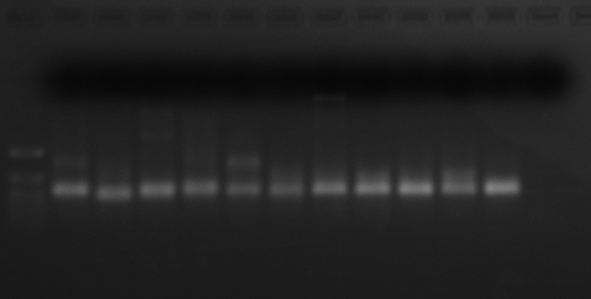

Supplement: Supplementary file 3 — Additional file 3. Uncropped gel picture annotated in Additional file 2: Figure S12a. [file 12915_2021_1104_MOESM3_ESM.tiff]
